# Characteristics of insulin resistance in Korean adults from the perspective of circadian and metabolic sensing genes

**DOI:** 10.1007/s13258-023-01443-0

**Published:** 2023-09-28

**Authors:** Miso S. Park, Siwoo Lee, Younghwa Baek, Juho Lee, Sang-Soo Park, Jung-Hyo Cho, Hee-Jeong Jin, Ho-Ryong Yoo

**Affiliations:** 1 Clinical Trial Center, Daejeon Korean Medicine Hospital of Daejeon University, 75 Daedeok-daero 176beon-gil, Seo- gu, Daejeon, 35235 Korea; 2 Department of Cardiology and Neurology of Korean Medicine, College of Korean Medicine, Daejeon University, Daejeon, Korea; 3 KM Data Division, Korea Institute of Oriental Medicine, 1672 Yuseong-daero, Yuseong-gu, Daejeon, 34054 Korea; 4 Data Convergence Drug Research Center, Korea Research Institute of Chemical Technology, University of Science & Technology, Daejeon, Korea; 5 Liver and Immunology Research Center, Daejeon Korean Medicine Hospital of Daejeon University, Daejeon, Korea

**Keywords:** Insulin resistance, Circadian rhythm, Single nucleotide polymorphism, Adiposity, Inflammation, Iron overload

## Abstract

**Background:**

The biological clock allows an organism to anticipate periodic environmental changes and adjust its physiology and behavior accordingly.

**Objective:**

This retrospective cross-sectional study examined circadian gene polymorphisms and clinical characteristics associated with insulin resistance (IR).

**Methods:**

We analyzed data from 1,404 Korean adults aged 30 to 55 with no history of cancer and cardio-cerebrovascular disease. The population was classified according to sex and homeostasis model assessment of insulin resistance (HOMA-IR) values. Demographics, anthropometric and clinical characteristics, and single nucleotide polymorphisms (SNPs) were analyzed with respect to sex, age, and HOMA-IR values. We used association rule mining to identify sets of SNPs from circadian and metabolic sensing genes that may be associated with IR.

**Results:**

Among the subjects, 15.0% of 960 women and 24.3% of 444 men had HOMA-IR values above 2. Most of the parameters differed significantly between men and women, as well as between the groups with high and low insulin sensitivity. Body fat mass of the trunk, which was significantly higher in insulin-resistant groups, had a higher correlation with high sensitivity C-reactive protein and hemoglobin levels in women, and alanine aminotransferase and aspartate aminotransferase levels in men. Homozygous minor allele genotype sets of SNPs rs17031578 and rs228669 in the *PER3* gene could be more frequently found among women with HOMA-IR values above 2 (p = .014).

**Conclusion:**

Oxidative stress enhanced by adiposity and iron overload, which may also be linked to *NRF2* and *PER3*-related pathways, is related to IR in adulthood. However, due to the small population size in this study, more research is needed.

**Supplementary Information:**

The online version contains supplementary material available at 10.1007/s13258-023-01443-0.

## Introduction

The circadian rhythm, which is generated by endogenous oscillators, forms the temporal structure of human physiology by regulating energy metabolism in accordance with the natural light and dark cycle. When the circadian rhythm, gut microbial activities, and geophysical environmental signals are out of sync, energy metabolism can be disrupted, and conditions like insulin resistance may develop (Stenvers et al. 2019). Insulin sensitivity in skeletal muscle is remotely modulated by light and Sirtuin 1 (SIRT1) dependent pathway (Aras et al. 2019). It has been reported that certain polymorphic variants of *SIRT1*, as well as other circadian genes such as Circadian Locomotor Output Cycles Kaput (*CLOCK*), Brain and Muscle Arnt-Like 1 (*BMAL1*, also known as aryl hydrocarbon receptor nuclear translocator-like 1, *ARNTL1*), Cryptochrome 1 (*CRY1*) may lead to insulin resistance and increase the risk of metabolic and mood disorders (Dong et al. 2011; Dashti et al. 2014; Kovanen et al. 2015; Angelousi et al. 2018).

Various genome-wide association studies (GWASs) and meta-analyses of GWASs have identified single nucleotide polymorphisms (SNPs) from various loci associated with insulin resistance and type 2 diabetes (Hong et al. 2014). However, identifying the genes associated with disease phenotypes in complex, multifactorial disorders can be more challenging compared to that in simple Mendelian diseases. It is generally thought that the combined effects of multiple genetic variants with small effect sizes may constitute such complex traits. However, the genome-wide significance threshold at the level of p < 5.0 × 10^− 8^, which is applied in GWAS to prevent type 1 error, might filter SNPs with small effect sizes unless the sample size is extremely large. Therefore, additional methodologies might be necessary in order to compensate for the limitations of traditional GWASs (Stringer et al. 2011).

Breuer et al. (2018) addressed such issues of concern for GWAS of complex traits and hypothesized that analyzing the joint effect of genetic variants via the association rule mining (ARM) technique could be an alternative solution. ARM technique is a data mining technique that aims to extract frequent patterns from large databases. The technique is generally applied to analyze customer habits from transaction databases, and gene expression from microarray data. Breuer et al. considered this technique can unravel unknown associations between genome-wide genetic variants. Besides the genetic variation of specific SNPs, minor allele content (MAC) can be calculated as a quantitative measurement. Genome-wide accumulation of MAC in individuals might play a role in the pathogenesis of complex diseases such as type 2 diabetes and Alzheimer’s disease (Lei and Huang 2017; Chen et al. 2020). According to Chen et al. (2014) and Kido et al. (2018), it can be hypothesized that as the result of a natural selection process, risk alleles, compared to protective alleles might face negative selection and be kept at lower proportions. Therefore, risk alleles are more likely to be found among minor alleles.

From the above literature, we have concluded that individuals who carry certain variants of circadian genes may be more vulnerable to metabolic disorders. For the circadian genes, considering the joint effects of multiple variants and MAC might further enhance the prediction power of genome-wide association models. In this study, we will examine sex differences in circadian gene polymorphisms, and demographic and clinical characteristics associated with insulin resistance. Our hypothesis is: carrying certain sets of homozygous minor alleles or more minor alleles in the circadian and metabolic sensing genes may be metabolically riskier in the long term thus contributing to the pathogenesis of insulin resistance.

## Materials and methods

### Study design and settings

In this retrospective cross-sectional study, we analyzed the data of Korean adults (n = 1404) provided by the Korean medicine Data Center (KDC). The study protocol conformed to the declaration of Helsinki. Due to the retrospective nature of the work, this study was granted an exemption by the local ethics committee with a waiver of consent (Daejeon University institutional review board exemption: 1040647-202104-HR-019-03). The study population were relatively healthy adults recruited from the local community from 2017 to 2019, and had complete data on demographics, vital signs, anthropometric and bioelectric impedance measurements, hematological profile, and single nucleotide polymorphisms (SNPs). All data were anonymized, and the SNP data were analyzed on-site at the data center using a computer completely isolated from the internet and secured with a password and a locking device.

### Study population

For this study, we used minimal inclusion and exclusion criteria so that the study population could be representative of the general population. The inclusion criterion was: relatively healthy adults recruited from the local community, who had complete data on demographics, vital signs, anthropometric and bioelectric impedance measurements, hematological profiles, and SNPs. The exclusion criterion was: those with a history of malignant tumor or cardio-cerebrovascular diseases.

### Hematological profile

Overnight fasting blood samples (about 22.5 ml) were collected between 8 AM and 12 PM from the median cubital vein of each participant. The samples were centrifuged for 10 min at 3450 rpm and then transported to the laboratory (Seoul Clinical Laboratories, Yongin, Korea) within 24 h of collection for hematological examination. The blood test includes complete blood count, liver function, lipid profile, fasting plasma glucose and insulin levels, inflammatory markers, and differential count. Granulocyte count was calculated by summing the neutrophil, eosinophil and basophil counts. The granulocyte to lymphocyte ratio (GLR) was calculated by dividing the granulocyte count by the lymphocyte count, the neutrophil to lymphocyte ratio (NLR) was calculated by dividing the neutrophil count by the lymphocyte count, and the lymphocyte to monocyte ratio (LMR) was calculated by dividing the monocyte count by the lymphocyte count.

In this study, we used the homeostasis model assessment of insulin resistance (HOMA-IR), an indirect measurement of insulin resistance. HOMA-IR was calculated from fasting blood insulin and glucose levels. The primary cut-off point used for HOMA-IR in this study was 2, which corresponds to about the 75th percentile in the Korean population aged 40–69 years in Kim et al. (2018)’s study. The formula used for calculating HOMA-IR was as follows:

$$\text{H}\text{O}\text{M}\text{A}-\text{I}\text{R} = \frac{\text{f}\text{a}\text{s}\text{t}\text{i}\text{n}\text{g} \text{g}\text{l}\text{u}\text{c}\text{o}\text{s}\text{e} (\text{m}\text{g}/\text{d}\text{L})\times \text{f}\text{a}\text{s}\text{t}\text{i}\text{n}\text{g} \text{i}\text{n}\text{s}\text{u}\text{l}\text{i}\text{n}(\text{u}\text{U}/\text{m}\text{L})}{405}$$

### Measurements

Anthropometric measurements were obtained and recorded according to a standardized protocol. Height, weight, waist and hip circumferences were measured to the nearest ± 0.1 cm and kg. Body mass index (BMI) was calculated as body weight in kilograms divided by the square of height in meters (kg/m^2^). Mass and proportion of body fat and muscle were measured with the InBody 770 bioelectrical impedance analyzer (Biospace, Seoul, Korea).

### Gene analysis

#### SNP selection

A total of 750,050 SNPs were analyzed using the Theragen Precision Medicine Research Array (PMRA) chip (TheragenEtex Bio Institute, Suwon, Korea). Following genes that are involved in the circadian cycle and metabolic sensing (Rijo-Ferreira and Takahashi 2019; Stenvers et al. 2019) were selected for this study: *AKT1, AKT2, ARNTL1, ARNTL2, CLOCK, CRY1, CRY2, DEC1, FOXO1, IRS1, JAK2, LIPE, LPL, MAPK8, NFE2L2, NFKB1, NPAS2, NR1D1, NR1D2, PBP4, PCK2, PER1, PER2, PER3, PNPLA2, RORA, RORB, RORC, SIRT1, SIRT6, SLC2A1, SLC2A2, SLC16A1, SLC16A4, SLC16A7, SREBF1, STAT3, STRA6*, and *TIMELESS*. A list of SNPs for each gene was downloaded from the Single-Nucleotide Polymorphism database (National Center for Biotechnology Information (NCBI) dbSNP). Only common, intersecting SNPs from the selected genes and the Theragen PMRA chip were used for the analysis. All SNPs were autosomal SNPs. Before data analysis, SNPs were pruned using PLINK (v1.07) software (Purcell et al. 2007). SNPs with minor allele frequency and Hardy-Weinberg equilibrium of greater than 0.05 were selected. Then, SNPs with missingness per marker greater than 10%, and missingness per individual greater than 5%, were excluded. This resulted in 478 SNPs that were passed on to later processes.

**Fig. 1:**
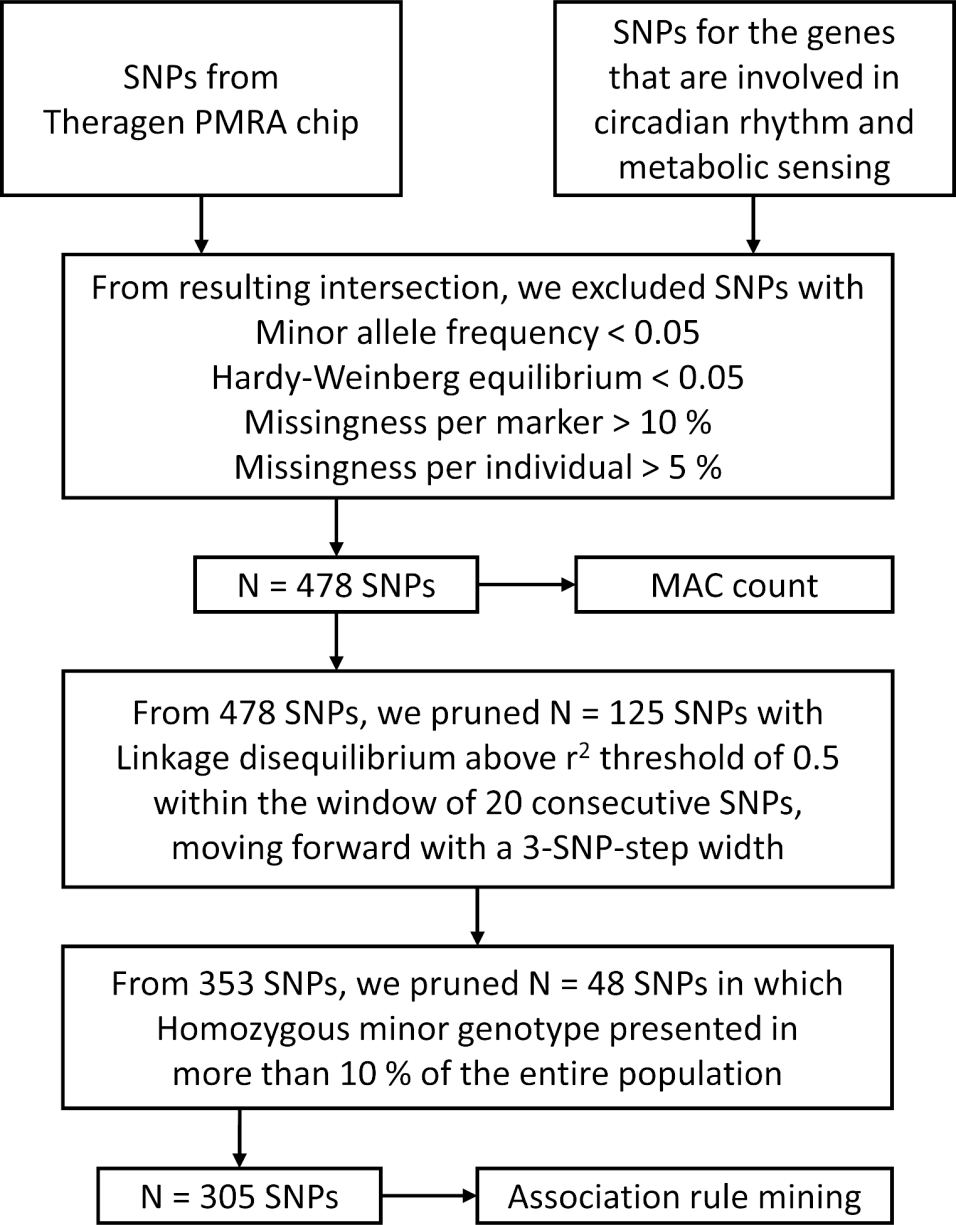
Schematic diagram of the SNP selection process for cluster analysis and exploratory association rule mining. SNP, single nucleotide polymorphism; PMRA, Precision Medicine Research Array; MAC, minor allele content

Linkage disequilibrium (LD) in a specific population refers to a non-random association of alleles at different loci. LDs for the SNPs used in this study were calculated using PLINK software. In this study, we pruned SNPs with LD above the r^2^ threshold of 0.5 with PLINK option --indep-pairwise 20 3 0.5. This left us with 353 SNPs that were passed on to later processes. Further processes for selecting and pruning SNPs are shown in Fig. 1.

#### Exploratory association rule mining

For association rule mining, if the input data are too scarce, then rules can hardly be mined. Meanwhile, if all the SNPs including those with higher MAF are included in the association rule mining, then the homozygous minor allele genotypes with higher MAF will have a higher chance to be randomly associated with each other compared to those with lower MAF. Since risk alleles, in many cases, tend to be minor alleles with low MAF (Kido et al. 2018), we had to find a point of compromise by further pruning process. Therefore, from the SNPs used above, those with high MAF, in which homozygous minor allele genotype presented in more than 10% of the entire population were removed.

Genotype data of the 305 SNPs from 1404 individuals were obtained with PLINK software. The Association rule mining algorithm was programmed with Python 3.9.1 (Python Software Foundation, Wilmington, DE, USA) using the Mlxtend library (Raschka 2018). For association rule mining, minor allele-minor allele pairs were coded as 1 and minor allele-major allele pairs and major allele-major allele pairs were all coded as 0. All missing values in genotype data were assumed to be non-homozygous minor genotypes and thus were coded as 0. We created dummy variables and dropped the minor-major and major-major allele categories so that we could come up with association rules between homozygous minor allele genotypes as a result.

We divided the SNP dataset according to males and females with high and normal HOMA-IR values. From the entire dataset of *n* items, we can generate $\sum _{k=2}^{n}\left(\genfrac{}{}{0pt}{}{n}{k}\right)\times ({2}^{k}-2)$ association rules. We then applied the apriori algorithm to the dataset to obtain candidates for the later association rule mining step. We used minimum support of 0.05 which is how often the item is present in the whole dataset. For the association rules, we used a threshold of 0.1 for minimum confidence. For the rules consisting of SNP sets of A and B, the support S is defined as follows: $S\left(A\to B\right)=P\left(A\cap B\right)=N(A\cap B)/n$, where *P* stands for the probability and *N* stands for the number of sets. The support is used to select the frequently occurring sets from the entire dataset. Meanwhile, the confidence C is defined as follows: $C\left(A\to B\right)=P\left(B|A\right)=N(A\cap B)/N\left(A\right)$. The confidence, in this case, is the probability of SNP B appears given the SNP A appears. The association rule mining algorithm looks for sets with a high level of support and confidence in relation to their threshold.

#### Minor allele content

MAC of each individual was counted from the above dataset of n = 478 SNPs for circadian genes, and n = 133 SNPs from core clock genes including *ARNTL1, ARNTL2, CLOCK, CRY1, CRY2, DEC1, NPAS2, PER1, PER2*, and *PER3*. Furthermore, we pruned the SNPs using different MAFs of 0.1 or 0.3.

### Statistical analysis

The Hardy-Weinberg equilibrium, missing data, MAF, and LD were processed and calculated using PLINK software. The rest of the statistical analysis was performed using SPSS 23.0 for Windows (International Business Machines Corporation, Armonk, NY, USA) and Python 3.9.1 (Python Software Foundation, Wilmington, DE, USA). We applied log-normalization before analysis when appropriate. Males and females were analyzed separately. Differences were considered significant at P < .05. Correlation coefficients were interpreted as being negligible (0.00-0.10), weak (0.10–0.39), moderate (0.40–0.69), and strong (0.70 to 1.00).

## Results

### Population characteristics

Table 1 contains the demographic and clinical characteristics of the study population. The age of the population ranged from 30 to 55 years. Among the 1404 subjects, 960 (68.4%) were women and 444 (31.6%) were men. Most of the clinical characteristics differed significantly between men and women. Therefore, we conducted statistical analyses on men and women separately. Furthermore, because the number of male subjects was lower than that of female subjects, the statistical power of males in this study was lower than that of females. The mean ages of men and women were also found to be significantly different. The actual difference in the mean age, however, was about 1.5 years, so we considered that it was clinically negligible.

The distribution of HOMA-IR in men and women in this study population is shown in Table 2. HOMA-IR of 2, which was used as a cut-off point in this study, was at the 82nd percentile in the entire population, 85th percentile in women, and 76th percentile in men. Among the study population, 144 (15.0%) women and 108 (24.3%) men had HOMA-IR values above 2. There were significant differences in most of the clinical characteristics between the groups with HOMA-IR values above and below 2 (Table 1).

**Table 1:** Demographic and Clinical Characteristics of the Study Population

	Women				Men				All
	All (n = 960)	HOMA-IR < 2 (n = 816)	HOMA-IR **≥** 2 (n = 144)	P for t-test	All (n = 444)	HOMA-IR < 2 (n = 336)	HOMA-IR **≥** 2 (n = 108)	P for t-test	P for t-test
Age, yr	44.6 ± 6.6	44.7 ± 6.5	44.2 ± 7.0	0.428	43.1 ± 7.1	43.3 ± 7.0	42.6 ± 7.3	0.343	< .001^a^
BMI, kg/m^2^	23.9 ± 3.5	23.3 ± 2.9	27.7 ± 4.3	< .001^a^	25.8 ± 3.3	25.0 ± 2.8	28.5 ± 3.3	< 0.001 ^a^	< .001^a^
SBP, mm Hg	112.7 ± 15.2	111.5 ± 14.9	119.4 ± 15.1	< 0.001	123.6 ± 14.1	122.1 ± 14.0	128.0 ± 13.3	< 0.001	< 0.001
DBP, mm Hg	70.1 ± 11.4	69.2 ± 11.2	74.9 ± 11.1	< 0.001	81.0 ± 11.3	79.8 ± 11.0	84.7 ± 11.7	< 0.001	< 0.001
FPI, μIU/mL	5.9 ± 4.2	4.6 ± 1.9	13.0 ± 6.3	< .001^a^	7.0 ± 5.2	5.0 ± 2.2	13.3 ± 6.8	< 0.001 ^a^	< .001^a^
FPG, mg/dL	82.8 ± 13.9	80.7 ± 8.7	95.0 ± 26.1	< .001^a^	87.5 ± 20.6	83.2 ± 9.8	101.1 ± 34.8	< 0.001 ^a^	< .001^a^
HbA_1c_, %	5.4 ± 0.5	5.4 ± 0.4	5.9 ± 0.9	< .001^a^	5.6 ± 0.7	5.5 ± 0.4	6.0 ± 1.2	< 0.001 ^a^	< .001^a^
AST, IU/L	23.9 ± 12.8	22.8 ± 8.9	29.9 ± 24.7	.001^a^	28.8 ± 13.0	27.7 ± 10.2	32.4 ± 18.8	0.014 ^a^	< .001^a^
ALT, IU/L	20.8 ± 18.0	18.1 ± 10.2	35.5 ± 36.3	< .001^a^	34.2 ± 23.6	31.1 ± 20.1	43.8 ± 30.2	< 0.001 ^a^	< .001^a^
GGT, IU/L	22.9 ± 24.2	20.5 ± 20.3	36.4 ± 37.1	< .001^a^	56.1 ± 59.7	51.2 ± 56.4	71.4 ± 67.1	0.005 ^a^	< .001^a^
TG, mg/dL	110.9 ± 66.3	103.3 ± 59.0	153.9 ± 86.1	< .001^a^	189.7 ± 169.2	169.0 ± 152.0	254.3 ± 201.9	< 0.001 ^a^	< .001^a^
HDL-C, mg/dL	59.6 ± 13.8	61.2 ± 13.8	50.5 ± 10.7	< .001^a^	49.5 ± 10.8	51.1 ± 11.0	44.6 ± 8.5	< 0.001 ^a^	< .001^a^
LDL-C, mg/dL	118.0 ± 32.6	115.7 ± 31.4	131.1 ± 36.2	< 0.001	125.5 ± 33.0	123.9 ± 32.2	130.4 ± 35.0	0.069	< 0.001

Values are expressed as mean ± standard deviation.

HOMA-IR, homeostasis model assessment of insulin resistance, BMI, body mass index, SBP, systolic blood pressure, DBP, diastolic blood pressure, FPI, fasting plasma insulin, FPG, fasting plasma glucose, HbA_1c_, hemoglobin A1c, AST, aspartate aminotransferase, ALT, alanine aminotransferase, GGT, gamma-glutamyltransferase, TG, triglyceride, HDL-C, high-density lipoprotein cholesterol, LDL-C, low-density lipoprotein cholesterol.

^a^Mann-Whitney U test was used instead as the P-value of Levene’s test for equality of variances was less than 0.05.

**Table 2:** Distribution of HOMA-IR in Men and Women

	Percentile				
	10th	25th	50th	75th	90th
HOMA-IR (all)	0.48	0.68	1.05	1.63	2.41
HOMA-IR (women)	0.47	0.64	0.99	1.44	2.27
HOMA-IR (men)	0.50	0.76	1.16	1.93	2.80

HOMA-IR, homeostasis model assessment of insulin resistance

The hematological characteristics of the study population are shown in Table 3. Most of the hematological characteristics differed significantly between men and women, and between the groups with high and low insulin sensitivity (i.e., HOMA-IR value below and above 2). Subjects with a HOMA-IR value of 2 or higher had higher levels of high-sensitivity C-reactive protein (hsCRP), neutrophils, lymphocytes, monocytes, red blood cell (RBC) count, hemoglobin, and hematocrit, and lower mean corpuscular volume (MCV) and mean corpuscular hemoglobin (MCH) in both men and women. In addition, women with HOMA-IR 2 or higher had higher GLR and NLR.

**Table 3:** Hematological Characteristics of the Study Population

	Women				Men				All
	All (n = 960)	HOMA-IR < 2 (n = 816)	HOMA-IR **≥** 2 (n = 144)	P for t-test	All (n = 444)	HOMA-IR < 2 (n = 336)	HOMA-IR **≥** 2 (n = 108)	P for t-test	P for t-test
hsCRP, mg/L	1.3 ± 3.4	1.1 ± 3.2	2.6 ± 4.5	< .001^a^	1.2 ± 1.8	1.1 ± 1.8	1.6 ± 1.7	0.016	.404^a^
Neutrophils, /mm^3^	3140 ± 1220	3020 ± 1140	3780 ± 1450	< .001^a^	3400 ± 1190	3340 ± 1150	3610 ± 1310	0.047	< 0.001
Lymphocytes, /mm^3^	1780 ± 500	1750 ± 480	1980 ± 540	< 0.001	2060 ± 540	2030 ± 540	2160 ± 540	0.029	< .001^a^
Monocytes, /mm^3^	279 ± 84	272 ± 82	314 ± 90	< 0.001	338 ± 103	332 ± 103	356 ± 101	0.036	< .001^a^
RBC, 10^6^/μL	4.4 ± 0.3	4.3 ± 0.3	4.6 ± 0.3	< 0.001	5.0 ± 0.3	4.9 ± 0.3	5.1 ± 0.3	< 0.001	< 0.001
Hb	13.1 ± 1.2	13.0 ± 1.2	13.5 ± 1.2	< 0.001	15.4 ± 0.9	15.3 ± 0.9	15.6 ± 0.9	0.010	< 0.001 ^a^
Hct	40.6 ± 3.1	40.4 ± 3.0	41.8 ± 3.2	< 0.001	46.8 ± 2.8	46.7 ± 2.8	47.3 ± 2.7	0.035	< 0.001
MCV, fL	93.2 ± 6.1	93.4 ± 6.2	91.8 ± 5.4	0.004	94.2 ± 4.0	94.5 ± 4.0	93.3 ± 4.0	0.004	< 0.001 ^a^
MCH	30.1 ± 2.4	30.1 ± 2.5	29.8 ± 2.2	0.049	31.0 ± 1.3	31.0 ± 1.3	30.7 ± 1.3	0.023	< 0.001 ^a^
MCHC	32.3 ± 1.1	32.2 ± 1.1	32.4 ± 1.1	0.212	32.9 ± 0.8	32.8 ± 0.9	32.9 ± 0.8	0.411	< 0.001 ^a^
GLR	2.0 ± 0.9	1.9 ± 0.9	2.1 ± 0.9	0.020	1.9 ± 0.8	2.0 ± 0.9	2.0 ± 0.8	0.920	0.038
NLR	1.9 ± 0.8	1.8 ± 0.8	2.0 ± 0.8	0.022	1.7 ± 0.8	1.7 ± 0.8	1.7 ± 0.7	0.855	0.018
LMR	6.8 ± 2.2	6.8 ± 2.1	6.7 ± 2.7	0.925	6.5 ± 2.0	6.5 ± 2.0	6.4 ± 1.8	0.647	0.012

Values are expressed as mean ± standard deviation.

HOMA-IR, homeostasis model assessment of insulin resistance, hsCRP, high-sensitivity C-reactive protein, RBC, red blood cells, Hb, hemoglobin, Hct, hematocrit, MCV, mean corpuscular volume, MCH, mean corpuscular hemoglobin, MCHC, mean corpuscular hemoglobin concentration, GLR, granulocyte to lymphocyte ratio, NLR, neutrophil to lymphocyte ratio, LMR, lymphocyte to monocyte ratio.

^a^Mann-Whitney U test was used instead as the P-value of Levene’s test for equality of variances was less than 0.05.

The body impedance measurements of the study population are shown in Table 4. Compared to men, women had a greater visceral fat area (VFA), percentage of body fat (PBF), body fat mass (BFM) of the upper and lower limbs, fat to lean mass ratio (FLMR), fat to skeletal muscle mass ratio (FSMMR), and lean fat to trunk fat ratio (LFTFR). Meanwhile, men had greater soft lean mass (SLM) and skeletal muscle mass (SMM). BFM of the trunk did not differ between men and women. Subjects with HOMA-IR of 2 or higher had higher VFA, PBF, BFM, FLMR, SLM, SMM, FSMMR, and LFTFR.

**Table 4:** Clinical Differences in Fat and Muscle Distributions and Ratios Based on Body Impedance Measurements

	Women				Men				All
	All (n = 960)	HOMA-IR < 2 (n = 816)	HOMA-IR **≥** 2 (n = 144)	P for t-test	All (n = 444)	HOMA-IR < 2 (n = 336)	HOMA-IR **≥** 2 (n = 108)	P for t-test	P for t-test
VFA	100.6 ± 37.2	93.7 ± 31.9	139.5 ± 41.3	< .001^a^	87.2 ± 35.4	79.1 ± 30.7	112.5 ± 37.3	< .001^a^	< .001^a^
PBF, %	34.0 ± 6.0	33.1 ± 5.6	39.2 ± 5.5	< 0.001	25.8 ± 6.3	24.6 ± 6.1	29.7 ± 5.4	< 0.001	< 0.001
BFM, kg	20.8 ± 6.5	19.6 ± 5.2	28.0 ± 8.0	< .001^a^	20.0 ± 7.0	18.3 ± 6.1	25.2 ± 7.2	< .001^a^	0.022
Upper limbs, kg	1.5 ± 0.6	4.4 ± 1.9	2.8 ± 0.9	< .001^a^	1.3 ± 0.7	2.2 ± 1.1	3.5 ± 1.6	< .001^a^	< 0.001
Lower limbs, kg	3.2 ± 0.9	6.1 ± 1.5	8.2 ± 2.2	< .001^a^	2.8 ± 0.9	5.3 ± 1.5	6.9 ± 1.8	< .001^a^	< 0.001
Trunk, kg	10.3 ± 3.3	9.7 ± 2.8	14.0 ± 3.8	< .001^a^	10.5 ± 3.8	9.6 ± 3.4	13.4 ± 3.8	< .001^a^	.381^a^
SLM, kg	37.1 ± 4.4	36.6 ± 4.1	40.1 ± 4.9	< .001^a^	53.0 ± 6.8	52.1 ± 6.5	55.5 ± 7.0	< 0.001	< .001^a^
SMM, kg	21.3 ± 2.8	20.9 ± 2.6	23.2 ± 3.1	< .001^a^	31.6 ± 4.3	31.0 ± 4.2	33.2 ± 4.4	< 0.001	< .001^a^
FLMR	0.561 ± 0.150	0.537 ± 0.134	0.696 ± 0.161	< .001^a^	0.379 ± 0.130	0.354 ± 0.123	0.455 ± 0.120	< 0.001	< .001^a^
FSMMR	0.980 ± 0.260	0.940 ± 0.236	1.203 ± 0.276	< 0.001	0.637 ± 0.221	0.596 ± 0.212	0.763 ± 0.203	< 0.001	< .001^a^
LFTFR	0.459 ± 0.036	0.922 ± 0.071	0.892 ± 0.066	< 0.001	0.414 ± 0.411	0.848 ± 0.944	0.770 ± 0.064	0.395	.023^a^

Values are expressed as mean ± standard deviation.

HOMA-IR, homeostasis model assessment of insulin resistance, VFA, visceral fat area, PBF, percent body fat, BFM, body fat mass, SLM, soft lean mass, SMM, skeletal muscle mass, FLMR, fat to lean mass ratio, FSMMR, fat to skeletal muscle mass ratio, LFTFR, lean fat to trunk fat ratio.

^a^Mann-Whitney U test was used instead as the P-value of Levene’s test for equality of variances was less than 0.05.

**Fig. 2:**
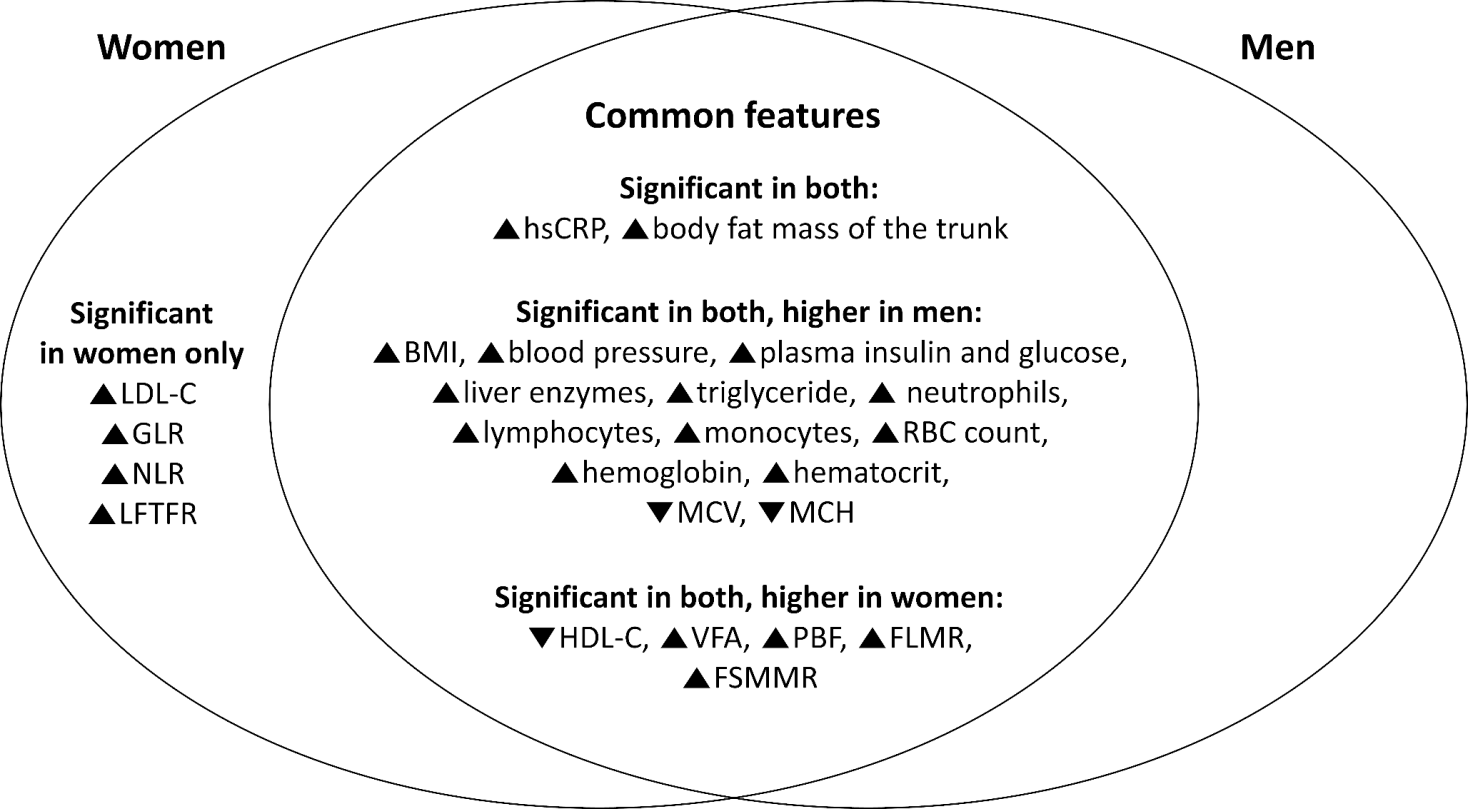
Shared and unique features of insulin resistance between men and women. LDL-C, low-density lipoprotein cholesterol; GLR, granulocyte to lymphocyte ratio; NLR, neutrophil to lymphocyte ratio; LFTFR, lean fat to trunk fat ratio; hsCRP, high sensitivity C-reactive protein; BMI, body mass index; RBC, red blood cell; MCV, mean corpuscular volume; MCH, mean corpuscular hemoglobin, HDL-C, high-density lipoprotein cholesterol; VFA, visceral fat area; PBF, percent body fat; FLMR, fat to lean mass ratio; FSMMR, fat to smooth muscle mass ratio

Figure 2 summarizes the contents in Tables 1 and 3, and 4. Men and Women in the study population demonstrated both similar and different pathophysiological features of insulin resistance. The shared pathophysiological features of insulin resistance in men and women were increased levels of hsCRP and BFM of the trunk. The features that were found significant only in insulin-resistant women were elevated levels of low-density lipoprotein cholesterol (LDL-C), GLR, NLR, and LFTFR. Meanwhile, BMI, blood pressure, fasting plasma insulin and glucose, liver enzymes, triglyceride, neutrophils, lymphocytes, monocytes, RBC count, hemoglobin, and hematocrit were all found to be significantly elevated in the insulin-resistant population and they were also significantly higher in men. VFA, PBF, FLMR, and FSMMR were significantly elevated in the insulin-resistant population and they were also significantly higher in women. Furthermore, correlations between levels of inflammatory markers, trunk fat mass, low-density lipoprotein cholesterol, liver enzymes, hemoglobin, and hematocrit in men and women are shown in Fig. S1. Correlations between HOMA-IR and clinical parameters in men and women in different age groups are shown in Table S13.

### Exploratory association rule mining

N = 283, 309, 143, 411 candidate rules were stratified for the group of (1) females and (2) males with high insulin sensitivity (normal HOMA-IR values), and (3) females and (4) males with low insulin sensitivity (high HOMA-IR values), respectively. The cut-off value of ≥ 2 was used to differentiate between normal and high HOMA-IR values. Supports ranged from 0.050 to 0.10 and confidence ranged from 0.46 to 1 in the outcome association rules.

Breuer et al. (2014) used binary variables in their work by coding homozygous minor alleles and heterozygous major-minor allele pairs as a single variable, and homozygous major alleles as another variable. Their resulting association rules consisted of sets of SNPs from different genes. However, in our study, we adopted a slightly different approach by coding homozygous minor alleles as one variable, and heterozygous minor-major allele pairs and homozygous major alleles into another variable, and dropped the latter variable when generating dummy variables. Therefore, in this study, we searched for the association patterns of homozygous minor alleles of the circadian and metabolic sensing genes. This resulted in the rules consisting of SNPs from the same genes only. It may be due to the relatively small sample of genes examined in this study, and linkage disequilibrium as proposed by Zondervan and Cardon (2004). Since some of the association rules were shared among all groups while other rules only appeared in certain groups, we compiled all the rules from groups with different clinical features altogether. Among the rules, those that were shared but displayed different frequencies between the groups (examined by the Chi-square test with a significance level of 0.10) are shown in Table 5. The characteristics of the SNPs in Table 5 are shown in Table 6. Considering the significance level of 0.05, only one set of SNPs in the *PER3* gene was significant. However, the statistical power of this study was low and this significance would vanish if we consider the Bonferroni correction. Detailed results are attached as supplementary data, Tables S1–12.

**Table 5:** Results for association rule mining on insulin sensitive and resistant men and women

Gene	SNP set	Insulin sensitive		Insulin resistant	
		Women (n = 816)	Men (n = 336)	Women (n = 144)	Men (n = 108)
*NFE2L2* (*NRF2*)	rs10930781, rs2364720, rs4243387	47(0.058) †	24(0.071)	14(0.097) †	11(0.10)
	rs10188107, rs10930781, rs4243387	43(0.053) †	23(0.068)	13(0.090) †	11(0.10)
	rs10188107, rs2364720, rs4243387	43(0.053) †	23(0.068)	13(0.090) †	11(0.10)
	rs10188107, rs10930781, rs2364720	43(0.053) †	23(0.068)	13(0.090) †	11(0.10)
*PER3*	rs17031578, rs228669	42(0.051) *	21(0.063)	15(0.10) *‡	N/A(< 0.05) ‡

Data are expressed as n(support).

The difference within the same sex, significant at * p < .05, † p < .1; difference between different sex, significant at # p < .05, ‡ p < .1 according to the Chi-square test. *NFE2L2*, nuclear factor erythroid-derived 2-like 2, *NRF2*, nuclear factor erythroid 2-related factor 2, *PER3*, period3.

**Table 6:** Position, MAF, and Variant Type of SNPs rs10930781, rs2364720, rs10188107, rs4243387, rs17031578, and rs228669 in *NFE2L2* and *PER3* genes

Gene	SNP	Position	Minor allele	Major allele	MAF	Variant type
*NFE2L2* (*NRF2*)	rs10930781	chr2:177249904	A	G	A = 0.2783	Intron variant
	rs2364720	chr2:177240416	A	G	A = 0.284	Intron variant
	rs10188107	chr2:177255584	T	G	A = 0.2549	Intron variant
	rs4243387	chr2:177253037	C	T	A = 0.2777	Intron variant
*PER3*	rs17031578	chr1:7799131	C	A	C = 0.2438	Intron variant
	rs228669	chr1:7809988	T	C	T = 0.2434	Synonymous variant

MAF, minor allele frequency; SNP, single nucleotide polymorphism; *NFE2L2*, nuclear factor erythroid-derived 2-like 2; *NRF2*, nuclear factor erythroid 2-related factor 2; *PER3*, period3.

Homozygous minor allele genotype sets of SNPs rs10930781, rs2364720, rs10188107, and rs4243387 (*NFE2L2* gene) and rs17031578 and rs228669 (*PER3* gene) could be more frequently found among females with HOMA-IR ≥ 2 (p = .072 for homozygous minor genotypes in SNP clusters of *NFE2L2* and p = .014 for that of *PER3*) although the estimated odds ratio for those variations were not high (1.8 for homozygous minor genotypes in SNP clusters of *NFE2L2* and 2.1 for that of *PER3* in women). Those sets of SNPs could be more frequently found among males with HOMA-IR ≥ 2 as well, although the results were not statistically significant.

### Effect of MAC in the pathophysiology of insulin resistance

MAC of specific genes for each individual was calculated as described in the methods and in Fig. 1. Pearson’s correlation analysis was performed between HOMA-IR and different MAC categories constructed using circadian genes and core clock genes as previously mentioned in the methods section. We performed the correlation analysis on groups of different ages and sex. Log-normalized HOMA-IR values and MAC categories did not show any correlation in female groups in their 30s, 40s, and 50s. Meanwhile, in males, different age groups showed different patterns of correlation between HOMA-IR values and MAC categories. For males in their 50s, homozygous minor/minor genotype count of core clock genes with MAF < 0.3 had a weak positive correlation (r = .2) with HOMA-IR values at the significance level of 0.05. For males in their 30 and 40 s, however, homozygous minor/minor genotype count of core clock genes with MAF < 0.3 was negatively correlated (r=-.12 without statistical significance, r=-.13 with marginal significance, respectively). The results are displayed in Table 7.

**Table 7:** Correlations (*r*) between minor allele content counts, homozygous minor/minor genotype counts and HOMA-IR values in men and women in different age groups

Population	Minor allele content (MAC) count				Homozygous minor/minor genotype count			
	Circadian genes, <.3^a^	Circadian genes, <.1^b^	Core clock genes, <.3^a^	Core clock genes, <.1^b^	Circadian genes, <.3^a^	Circadian genes, <.1^b^	Core clock genes, <.3^a^	Core clock genes, <.1^b^
All population	0.017	0.029	0.022	0.019	− 0.010	0.038	− 0.054*	− 0.007
Women (n = 960)								
30s (n = 242)	0.003	0.001	0.050	0.034	− 0.029	0.048	0.022	0.100
40s (n = 447)	0.040	0.018	0.039	0.000	0.042	0.060	− 0.068	− 0.049
50s (n = 271)	0.012	− 0.064	− 0.035	− 0.020	− 0.003	0.030	− 0.074	0.022
Men (n = 444)								
30s (n = 152)	0.055**	0.220	0.050	0.120	− 0.003	0.069	− 0.120	0.035
40s (n = 191)	− 0.008	0.079	− 0.001	0.041	− 0.081	− 0.024	− 0.130†	− 0.079
50s (n = 101)	− 0.066	0.047	0.015	0.092	− 0.069	0.110	0.200*	0.075

Pearson’s correlation coefficient (r) was significant at † p < .1, * p < .05, ** p < .01. We applied log-normalization before analysis when appropriate.

HOMA-IR, homeostasis model assessment of insulin resistance.

^a^<0.3: calculated from the SNPs with MAF below 0.3; ^b^<0.1: calculated from the SNPs with MAF below 0.1.

## Discussion

Insulin, an anabolic peptide hormone, is produced, stored, and secreted by the pancreatic beta cells in response to a glycemic stimulus. In healthy adults, β-cell responsiveness and insulin sensitivity are both higher in the morning and lower in the evening as they are under circadian control (Saad et al. 2012). In addition, plasma glucose and hepatic gluconeogenesis are also regulated by circadian metabolic rhythm (Simcox et al. 2015). Insulin resistance is a complex phenomenon that can be caused by diverse pathways. Proteomics studies revealed that there are different pathological processes of insulin resistance in skeletal muscle, adipose tissue, and liver (Li et al. 2020). Men and women have different body composition and physiology of fat and muscle, and the difference may lead to sex differences in the pathophysiology of insulin resistance. In general, women rely more on lipid metabolism while men tend to prefer utilizing glucose in a situation where energy is needed. Exercising induces a greater degree of lipolysis in women compared to that in men. Meanwhile, the carbohydrate oxidation rate is higher in men compared to that in women (Hedrington and Davis 2015). It might be due to the fact that the skeletal muscle is a major site for glucose uptake and decomposition. Men on average, compared to women, have greater skeletal muscle mass. It is well known that greater skeletal muscle mass is associated with increased insulin sensitivity while sarcopenia is known to be related to insulin resistance (Moon 2013).

In addition to metabolic preferences, lipid metabolism itself displays sexual dimorphism. For instance, the aquaglyceroporin (AQP) expression pattern is different in males and females. AQPs are channel protein that facilitates glycerol transportation across the cell membrane. AQPs are generally expressed in adipocytes and hepatocytes. In rodents, males have higher hepatic AQP9 protein, and thus greater hepatic glycerol permeability. Similar tendencies are observed in humans as men have higher hepatic glycerol permeability compared to women (Rodríguez et al. 2015). In this study, serum levels of liver enzymes were significantly higher in men and in subjects with HOMA-IR values above 2. In addition, there were stronger correlations between BFM of the trunk and ALT and AST in men compared to those in women. It could be possible that in insulin-resistant men, hepatic cell damage and the resulting oxidative stress and inflammation could be more prominent. In a community-based study by Cho et al., an increase in liver enzyme activities was accompanied by an increase in C-reactive protein in diabetic patients, and both of them were independent predictors for type 2 diabetes (Cho et al. 2007). In men, the accumulation of fat in the liver, central obesity, and inflammation may be one of the common pathophysiologies of insulin resistance and type 2 diabetes. These results may partially explain the higher incidence of fatty liver disease in men as well (Rodríguez et al. 2015).

On the other hand, the accumulation of body fat is related to chronic inflammatory conditions (Fernández-Sánchez et al. 2011). In this study, women, on average, had a higher percentage of body fat and FLMR compared to those of men. Parameters such as LDL-C, GLR, and NLR were elevated and LFTFR was decreased in insulin-resistant women but not in insulin-resistant men. In addition, insulin-resistant women had a higher level of hsCRP in their plasma compared to insulin-resistant men. There were stronger correlations between hsCRP and NLR and BFM of the trunk in women compared to those in men (Fig. S1). From these results, it could be postulated that women are more prone to systemic inflammation and oxidative stress due to higher adiposity.

Besides adiposity, elevated RBC count, hemoglobin levels and hematocrit were all related to insulin resistance in this study population. Meanwhile, hsCRP was weakly correlated with Hb and Hct in women only. Many studies have suggested that insulin has an erythropoietic effect and insulin receptor is engaged in RBC proliferation. It was previously described by Choi et al. that increased erythropoiesis and chronic subclinical inflammation are related to insulin resistance in elderly Koreans (Choi et al. 2003). Iron is a major source of hydroxyl radical (•OH) which can cause a wide range of biological damage. Insulin resistance is associated with iron overload as insulin can stimulate ferritin synthesis and facilitate iron uptake by cells. It has been recently discovered that iron overload can predict early death in a dose-dependent manner in the general population (Fernández-Real et al. 2015). Prolonged iron overload can lead to decreased lysosomal pools, autophagy failure, glucose intolerance and insulin resistance. Interventions such as an iron restriction diet, blood-letting, and chelation therapy could all increase insulin sensitivity (Jahng et al. 2019).

From the exploratory association rule mining, we could see that sets consisting of homozygous minor allele genotypes of SNPs rs10930781, rs2364720, rs10188107, and rs4243387 (from *NFE2L2* gene) and rs17031578 and rs228669 (from *PER3* gene) could be more frequently found among females with HOMA-IR ≥ 2 although the estimated odds ratio for those variations (1.8 for homozygous minor genotypes in SNP clusters of *NFE2L2* and 2.1 for that of *PER3*; all odds ratios may be applied to women only) are not that high. SNPs rs10930781, rs2364720, rs10188107, and rs4243387 are all intron variants of the *NFE2L2* gene. They have been reported in the studies on acute respiratory distress syndrome and adult-onset cognitive deficits induced by Phencyclidine as possible functional variants in the *NFE2L2* gene (Acosta-Herrera et al. 2015; Shirai et al. 2015) NFE2L2 (Nuclear factor-erythroid 2 like, also known as NRF2) is a transcription factor that regulates redox metabolism in cells. NFE2L2 activity increases during various stressful situations, including inflammation and redox perturbation to direct metabolic reprogramming of the cells and protect cells from oxidative damage. NFE2L2 is necessary for antioxidant function and nicotinamide adenine dinucleotide phosphate (NADPH) production in cells and tissues (Hayes and Dinkova-Kostova 2014; Uruno et al. 2015). In addition, NFE2L2 can regulate circadian rhythm and integrate redox metabolism into the tissue-specific circadian cycle by binding to enhancer regions of *CRY2* and the stress response element (STRE) motif of *PER3* and regulating their expression (Wible et al. 2018).

The cytoprotective and antioxidative activity of Kelch Like ECH Associated Protein 1 (KEAP1)-NRF2 system is important for glucose homeostasis as it can protect pancreatic beta cells from oxidative damage. Moreover, NRF2 plays an important role in iron metabolism. Iron can mediate glucose metabolism at multiple levels, and increased iron load can promote oxidative stress via the Fenton reaction (Kerins and Ooi 2018). NRF2 can restrict inflammation and protect cells from oxidative damage by regulating the pentose phosphate pathway (PPP). The PPP is also important for glutathione reduction, NADPH production, and nucleic acid biosynthesis (Early et al. 2018). Therefore, dysregulation of *NRF2* may render cells vulnerable to oxidative stress and chronic inflammation. *NRF2* can be a potential target for the treatment of chronic inflammation, insulin resistance, and type 2 diabetes (David et al. 2017). It has been recently discovered that *Nrf2* can restore leptin and insulin sensitivity by reducing the oxidative stress produced by the hypothalamus in mice (Yagishita et al. 2017).

Meanwhile, rs228669 SNP in the *PER3* gene is a synonymous variant while rs17031578 is an intron variant. In Kovac et al.’s study, SNP rs228669 was significantly associated with increased levels of triglycerides (TG) and risk of pre-term birth (Kovac et al. 2019). In Zhang et al.’s study, rs228669 SNP was significantly associated with the overall survival of patients with hepatocellular carcinoma. SNP rs228669 is located in the exonic splicing enhancer (ESE) region. Therefore, when there is a variation, it is possible that the sequence of mRNA, and structures and functions of the product protein can be altered (Zhang et al. 2014). Although it is not directly related to those SNPs above, *PER3* length polymorphism in its exon 18 has been reported in patients with type 2 diabetes (Karthikeyan et al. 2014). In addition, the transcript level of *PER3* was reduced in type 2 diabetes patients (Ando et al. 2009). It has been recently discovered that adipogenesis is regulated by the circadian rhythm generated by *Per3* in adipocyte precursor cells (APCs), and deletion of the *Per3* gene is related to increased adipogenesis in vivo in mice. In APCs in mice, the *Per3*-*Bmal1* complex can regulate adipogenesis by modulating *Klf15* (Kruppel-like factor 15) and *Pparγ* (peroxisome proliferator-activated receptor γ). Meanwhile, in human cells, there are conflicting results. Some studies reported that human *PER3* repressed adipogenesis in human mesenchymal stem cells and downregulation of human *PER3* immortalized bone marrow-derived Scp-1 cells and patient adipose-derived stromal cells. On the other hand, *PER3* enhanced adipogenesis in human adipose tissue-derived stromal cells (Aggarwal et al. 2017; Wan et al. 2021). Further research may be necessary to clarify the relationship of the *PER3* variations to the development of insulin resistance in humans.

Recently, Jakubiak et al. (2021) discovered that obesity and insulin resistance had the strongest relationship with oxidative stress among metabolic syndrome components such as “obesity and insulin resistance,” “dyslipidemia,” and “blood pressure.” It is worth noting that *NRF2* and *PER3* are related to the redox cycle and adipogenesis. According to Rey et al. (2016), inhibiting the PPP can alter circadian rhythm through mechanisms independent of the transcription-translation feedback loop and NRF2 is a key mediator linking the redox signals and circadian oscillations. 6-aminonicotinamide (6AN) treatment, which inhibits PPP and NADPH production, could delay the circadian phase in a reversible manner. Circadian genes, including *PER3*, displayed phase delays in transcription profiles as well. 6AN treatment also induced NRF2 activation and subsequent *PER3* response. NRF2 has been shown to bind to the STRE site and mediate *Per3* expression (Wang et al. 2012).

Meanwhile, the roles of *NRF2* and *PER3* in human adipogenesis still remain uncertain. Despite the fact that evidence from the majority of studies suggests that oxidative stress can upregulate Nrf2, which in turn promotes adipogenesis. Nrf2 inhibition can reduce lipid accumulation in response to oxidative stress. However, conflicting evidence suggests that Nrf2 can inhibit adipogenesis through the aromatic receptor pathway. More research is needed to clarify the pathway and fully comprehend the relationship between circadian rhythm, oxidative stress, obesity, and insulin resistance.

For the MAC of the circadian genes, it was found that homozygous minor genotype count of the circadian genes with MAF below < 0.3 were significantly correlated with HOMA-IR only in men in their 50s, although the correlation was very modest (r = .200, p < .05). For women, MAC or homozygous minor genotype count of the circadian genes did not display any significant correlation with HOMA-IR. It was interesting to note that homozygous minor/minor genotype count for the core clock genes with MAF < 0.03 was negatively correlated with HOMA-IR in men in their 30s (r=-.12) and 40s (r=-.13). However, for men in their 50s, the variables were positively correlated (r = .2). It might be due to aging-associated changes in gene expression. Further investigation is required. In a previous study conducted on the British population, it was found that enrichment of minor alleles of SNPs was found in patients with type 2 diabetes (Lei and Huang 2017). SNPs from genes other than circadian and metabolic sensing genes may need to be included in the model to enhance the predictive capability of MAC for insulin resistance and type 2 diabetes.

The goal of this study was to discover the minor alleles that might be related to the pathogenesis of insulin resistance. From this study, we have found that body composition and physiological differences in men and women may lead to sex differences in the pathophysiology of insulin resistance. Insulin-resistant women, compared to insulin-resistant men, had a greater degree of systemic inflammation and adiposity. Meanwhile, insulin-resistant men had greater levels of liver enzymes, plasma insulin and glucose, and hematological parameters such as hematocrit compared to those in insulin-resistant women.

From association rule mining results, we could find that homozygous minor allele contents in a circadian gene such as *PER3* may predispose individuals to be more prone to insulin resistance although additional evidence from further research is necessary. In this study, homozygous minor alleles of *PER3* could be more frequently found among women with decreased insulin sensitivity (p = .014). It may be due to the fact that women tend to rely more on lipid metabolism and alterations in the *PER3* gene may affect lipid metabolism. Meanwhile, homozygous minor alleles of *NFE2L2* could also be more frequently found among women with insulin resistance, although the result was not statistically significant (p = .072). The degree of systemic inflammation measured with hsCRP, GLR, and NLR was greater in insulin-resistant women compared to that in insulin-resistant men. Due to the higher level of systemic low-grade inflammation, women with decreased insulin sensitivity may be more prone to oxidative stress. However, because the number of female subjects was greater than that of male subjects, females had greater statistical power in this study than males. As a result, the greater significance observed in female groups could be a result of the power difference as well.

To conclude, oxidative stress due to adiposity and iron overload is related to insulin resistance in early to middle adulthood. Homozygous minor alleles of genes such as *PER3* and MAC of the circadian genes may predispose individuals to insulin resistance. However, the population in this research may be relatively too young and small to draw a meaningful conclusion. Therefore, further investigation of a larger population is necessary.

### Limitations

There are several limitations to this study. First, since the number of subjects was not large, our data have to be interpreted with careful consideration. We did not apply multiple comparison corrections for gene analysis because we only applied planned comparisons on specific SNPs. The results may no longer be significant if we apply Bonferroni correction. In this study, we primarily used the PLINK program for data processing and association rule mining for the main analysis. The reason for this is that the number of subjects was small and insufficient to perform GWAS. Rather than searching for SNPs associated with insulin resistance throughout the entire pool, the authors focused on the genes associated with the mechanism of insulin resistance and then looked for association rules within the SNPs of those genes. Searching for SNP sets rather than individual SNPs will also result in a larger effect size. Duplication studies with a larger population may be necessary to further verify and supplement the present investigation. In addition, there were fewer men compared to women in this study. So, the power of statistical tests may differ between men and women. The age of the population of this study is 30 to 55, which may be relatively young compared to other similar studies. Further research on the older population is necessary to apply the results to the population older than 55 years. Finally, we did not check the smoking status, drug history, and sleep and dietary patterns of the subjects in this study, which might have affected the health status of the participants. Further studies may be needed on those aspects as well.

### Electronic supplementary material

Below is the link to the electronic supplementary material.

Supplementary Material 1
