# Correction: characteristics of insulin resistance in Korean adults from the perspective of circadian and metabolic sensing genes

**DOI:** 10.1007/s13258-024-01493-y

**Published:** 2024-02-07

**Authors:** Miso S. Park, Siwoo Lee, Younghwa Baek, Juho Lee, Sang-Soo Park, Jung-Hyo Cho, Hee-Jeong Jin, Ho-Ryong Yoo

**Affiliations:** 1 Clinical Trial Center, Daejeon Korean Medicine Hospital of Daejeon University, 75 Daedeok-daero 176beon-gil, Seo- gu, Daejeon, 35235 Korea; 2 Department of Cardiology and Neurology of Korean Medicine, College of Korean Medicine, Daejeon University, Daejeon, Korea; 3 KM Data Division, Korea Institute of Oriental Medicine, 1672 Yuseong-daero, Yuseong-gu, Daejeon, 34054 Korea; 4 Data Convergence Drug Research Center, Korea Research Institute of Chemical Technology, University of Science & Technology, Daejeon, Korea; 5 Liver and Immunology Research Center, Daejeon Korean Medicine Hospital of Daejeon University, Daejeon, Korea

**Genes & Genomics (2023) 45:1475-1487**

10.1007/s13258-023-01443-0

In this article Miso S. Park was incorrectly denoted as the corresponding author but it should have been Hee-Jeong Jin; and Ho-Ryong Yoo;

