# Supplementary material for: Characteristics of insulin resistance in Korean adults from the perspective of circadian and metabolic sensing genes
Source: Genes Genomics. 2023 Sep 28;45(12):1475–87. doi: 10.1007/s13258-023-01443-0 (PMC10682234; doi:10.1007/s13258-023-01443-0)
Supplement: Supplementary file 1 — Supplementary Material 1 [file 13258_2023_1443_MOESM1_ESM.pdf]

Supplementary data

Table S1 Association rule mining results 1

SNP set	Normal		Insulin resistance	
	Female (n=816)	Male (n=336)	Female (n=144)	Male (n=108)
LPL_rs13702, LPL_rs326	N/A<br>Support < .05	N = 17<br>Support = .051	N/A<br>Support < .05	N = 8<br>Support = .074
IRS1_rs13431179, IRS1_rs16822642, IRS1_rs1801123	N/A<br>Support < .05	N = 19<br>Support = .057	N/A<br>Support < .05	N = 10<br>Support = .093
LPL_rs13702, LPL_rs15285, LPL_rs320	N/A<br>Support < .05	N = 17<br>Support = .051	N/A<br>Support < .05	N = 8<br>Support = .074
IRS1_rs13431179, IRS1_rs16822642	N/A<br>Support < .05	N = 19<br>Support = .057	N/A<br>Support < .05	N = 11<br>Support = .10
LPL_rs13702, LPL_rs15285, LPL_rs326	N/A<br>Support < .05	N = 17<br>Support = .051	N/A<br>Support < .05	N = 8<br>Support = .074
LPL_rs13702, LPL_rs15285	N/A<br>Support < .05	N = 17<br>Support = .051	N/A<br>Support < .05	N = 8<br>Support = .074
IRS1_rs13431554, IRS1_rs16822574	N/A<br>Support < .05	N = 18<br>Support = .054	N/A<br>Support < .05	N = 7<br>Support = .065
LPL_rs13702, LPL_rs320	N/A<br>Support < .05	N = 17<br>Support = .051	N/A<br>Support < .05	N = 8<br>Support = .074
LPL_rs13702, LPL_rs320, LPL_rs326	N/A<br>Support < .05	N = 17<br>Support = .051	N/A<br>Support < .05	N = 8<br>Support = .074
IRS1_rs13431179, IRS1_rs1801123	N/A<br>Support < .05	N = 19<br>Support = .057	N/A<br>Support < .05	N = 11<br>Support = .10

Table S2 Association rule mining results 2

SNP set	Normal		Insulin resistance	
	Female (n=816)	Male (n=336)	Female (n=144)	Male (n=108)
IRS1_rs13431179, IRS1_rs13431554, IRS1_rs16822574	N/A<br>Support < .05	N/A<br>Support < .05	N/A<br>Support < .05	N = 6<br>Support = .056
IRS1_rs10205233, IRS1_rs16822574	N/A<br>Support < .05	N/A<br>Support < .05	N/A<br>Support < .05	N = 6<br>Support = .056
LPL_rs13702, LPL_rs291	N/A<br>Support < .05	N/A<br>Support < .05	N/A<br>Support < .05	N = 8<br>Support = .074
LPL_rs13702, LPL_rs295, LPL_rs320	N/A<br>Support < .05	N/A<br>Support < .05	N/A<br>Support < .05	N = 8<br>Support = .074
LPL_rs13702, LPL_rs301, LPL_rs326	N/A<br>Support < .05	N/A<br>Support < .05	N/A<br>Support < .05	N = 8<br>Support = .074
LPL_rs13702, LPL_rs301, LPL_rs320	N/A<br>Support < .05	N/A<br>Support < .05	N/A<br>Support < .05	N = 8<br>Support = .074
IRS1_rs13431179, IRS1_rs16822574	N/A<br>Support < .05	N/A<br>Support < .05	N/A<br>Support < .05	N = 6<br>Support = .056
IRS1_rs10205233, IRS1_rs13431554, IRS1_rs16822574	N/A<br>Support < .05	N/A<br>Support < .05	N/A<br>Support < .05	N = 6<br>Support = .056
LPL_rs13702, LPL_rs15285, LPL_rs295	N/A<br>Support < .05	N/A<br>Support < .05	N/A<br>Support < .05	N = 8<br>Support = .074
LPL_rs13702, LPL_rs15285, LPL_rs291	N/A<br>Support < .05	N/A<br>Support < .05	N/A<br>Support < .05	N = 8<br>Support = .074
IRS1_rs10205233, IRS1_rs13431179, IRS1_rs16822574	N/A<br>Support < .05	N/A<br>Support < .05	N/A<br>Support < .05	N = 6<br>Support = .056
LPL_rs13702, LPL_rs295, LPL_rs301	N/A<br>Support < .05	N/A<br>Support < .05	N/A<br>Support < .05	N = 8<br>Support = .074

LPL_rs13702, LPL_rs291, LPL_rs326	N/A<br>Support < .05	N/A<br>Support < .05	N/A<br>Support < .05	N = 8<br>Support = .074
LPL_rs13702, LPL_rs291, LPL_rs295	N/A<br>Support < .05	N/A<br>Support < .05	N/A<br>Support < .05	N = 8<br>Support = .074
LPL_rs13702, LPL_rs291, LPL_rs301	N/A<br>Support < .05	N/A<br>Support < .05	N/A<br>Support < .05	N = 8<br>Support = .074
IRS1_rs13431179, IRS1_rs1560252, IRS1_rs16822642	N/A<br>Support < .05	N/A<br>Support < .05	N/A<br>Support < .05	N = 6<br>Support = .056
IRS1_rs10205233, IRS1_rs13431179, IRS1_rs13431554	N/A<br>Support < .05	N/A<br>Support < .05	N/A<br>Support < .05	N = 6<br>Support = .056
IRS1_rs10205233, IRS1_rs13431554	N/A<br>Support < .05	N/A<br>Support < .05	N/A<br>Support < .05	N = 6<br>Support = .056
LPL_rs13702, LPL_rs15285, LPL_rs301	N/A<br>Support < .05	N/A<br>Support < .05	N/A<br>Support < .05	N = 8<br>Support = .074
IRS1_rs1560252, IRS1_rs16822642	N/A<br>Support < .05	N/A<br>Support < .05	N/A<br>Support < .05	N = 6<br>Support = .056
IRS1_rs10205233, IRS1_rs13431179	N/A<br>Support < .05	N/A<br>Support < .05	N/A<br>Support < .05	N = 6<br>Support = .056
LPL_rs13702, LPL_rs301	N/A<br>Support < .05	N/A<br>Support < .05	N/A<br>Support < .05	N = 8<br>Support = .074
LPL_rs13702, LPL_rs295, LPL_rs326	N/A<br>Support < .05	N/A<br>Support < .05	N/A<br>Support < .05	N = 8<br>Support = .074
IRS1_rs13431179, IRS1_rs1560252, IRS1_rs1801123	N/A<br>Support < .05	N/A<br>Support < .05	N/A<br>Support < .05	N = 7<br>Support = .065
IRS1_rs1560252, IRS1_rs16822642, IRS1_rs1801123	N/A<br>Support < .05	N/A<br>Support < .05	N/A<br>Support < .05	N = 6<br>Support = .056
IRS1_rs13431179, IRS1_rs13431554	N/A<br>Support < .05	N/A<br>Support < .05	N/A<br>Support < .05	N = 6<br>Support = .056
LPL_rs13702, LPL_rs295	N/A<br>Support < .05	N/A<br>Support < .05	N/A<br>Support < .05	N = 8<br>Support = .074
RORA_rs2414682, RORA_rs4774376	N/A<br>Support < .05	N/A<br>Support < .05	N/A<br>Support < .05	N = 6<br>Support = .056
IRS1_rs1560252, IRS1_rs1801123	N/A<br>Support < .05	N/A<br>Support < .05	N/A<br>Support < .05	N = 7<br>Support = .065
LPL_rs13702, LPL_rs291, LPL_rs320	N/A<br>Support < .05	N/A<br>Support < .05	N/A<br>Support < .05	N = 8<br>Support = .074
IRS1_rs13431179, IRS1_rs1560252	N/A<br>Support < .05	N/A<br>Support < .05	N/A<br>Support < .05	N = 7<br>Support = .065

Table S3 Association rule mining results 3

SNP set	Normal		Insulin resistance	
	**Female (n=816)**	**Male (n=336)**	**Female (n=144)**	**Male (n=108)**
DEC1_rs10817745, DEC1_rs10982725	N = 41<br>Support = .050	N/A<br>Support < .05	N = 8<br>Support = .056	N/A<br>Support < .05

Table S4 Association rule mining results 4

SNP set	Normal		Insulin resistance	
	**Female (n=816)**	**Male (n=336)**	**Female (n=144)**	**Male (n=108)**
SLC2A2_rs11924032, SLC2A2_rs28720688	N/A<br>Support < .05	N/A<br>Support < .05	N = 10<br>Support = .069	N/A<br>Support < .05
SLC2A2_rs11924032, SLC2A2_rs7356034, SLC2A2_rs8192675	N/A<br>Support < .05	N/A<br>Support < .05	N = 9<br>Support = .063	N/A<br>Support < .05

NPAS2_rs3754675, NPAS2_rs72627425	N/A<br>Support < .05	N/A<br>Support < .05	N = 10<br>Support = .069	N/A<br>Support < .05
SLC2A2_rs11924032, SLC2A2_rs8192675	N/A<br>Support < .05	N/A<br>Support < .05	N = 9<br>Support = .063	N/A<br>Support < .05
SLC2A2_rs11924032, SLC2A2_rs28720688, SLC2A2_rs8192675	N/A<br>Support < .05	N/A<br>Support < .05	N = 9<br>Support = .063	N/A<br>Support < .05
RORA_rs4775287, RORA_rs999449	N/A<br>Support < .05	N/A<br>Support < .05	N = 9<br>Support = .063	N/A<br>Support < .05
SLC2A2_rs11924032, SLC2A2_rs28720688, SLC2A2_rs7356034	N/A<br>Support < .05	N/A<br>Support < .05	N = 10<br>Support = .069	N/A<br>Support < .05
SLC2A2_rs11924032, SLC2A2_rs7356034	N/A<br>Support < .05	N/A<br>Support < .05	N = 10<br>Support = .069	N/A<br>Support < .05

Supplementary S5 Association rule mining results 5

SNP set	Normal		Insulin resistance	
	**Female (n=816)**	**Male (n=336)**	**Female (n=144)**	**Male (n=108)**
RORA_rs7177996, RORA_rs7182392	N = 43<br>Support = .053	N/A<br>Support < .05	N/A<br>Support < .05	N/A<br>Support < .05
ARNTL1_rs11022759, ARNTL1_rs7130064	N = 42<br>Support = .051	N/A<br>Support < .05	N/A<br>Support < .05	N/A<br>Support < .05
NPAS2_rs17020663, NPAS2_rs57069400	N = 45<br>Support = .055	N/A<br>Support < .05	N/A<br>Support < .05	N/A<br>Support < .05

Table S6 Association rule mining results 6

SNP set	Normal		Insulin resistance	
	**Female (n=816)**	**Male (n=336)**	**Female (n=144)**	**Male (n=108)**
JAK2_rs3780381, JAK2_rs4495487	N = 41<br>Support = .050	N = 19<br>Support = .057	N/A<br>Support < .05	N/A<br>Support < .05
JAK2_rs3780381, JAK2_rs3824433, JAK2_rs4495487	N = 41<br>Support = .050	N = 19<br>Support = .057	N/A<br>Support < .05	N/A<br>Support < .05
JAK2_rs3780381, JAK2_rs3824433	N = 41<br>Support = .050	N = 19<br>Support = .057	N/A<br>Support < .05	N/A<br>Support < .05
JAK2_rs3824433, JAK2_rs4495487	N = 41<br>Support = .050	N = 19<br>Support = .057	N/A<br>Support < .05	N/A<br>Support < .05

Table S7 Association rule mining results 7

SNP set	Normal		Insulin resistance	
	**Female (n=816)**	**Male (n=336)**	**Female (n=144)**	**Male (n=108)**
SLC2A2_rs7356034, SLC2A2_rs8192675	N/A<br>Support < .05	N/A<br>Support < .05	N = 9<br>Support = .063	N = 6<br>Support = .056
SLC2A2_rs28720688, SLC2A2_rs8192675	N/A<br>Support < .05	N/A<br>Support < .05	N = 9<br>Support = .063	N = 6<br>Support = .056
SLC2A2_rs28720688, SLC2A2_rs7356034, SLC2A2_rs8192675	N/A<br>Support < .05	N/A<br>Support < .05	N = 9<br>Support = .063	N = 6<br>Support = .056
SLC2A2_rs28720688, SLC2A2_rs7356034	N/A<br>Support < .05	N/A<br>Support < .05	N = 10<br>Support = .063	N = 7<br>Support = .065

Table S8 Association rule mining results 8

SNP set	Normal		Insulin resistance	
	**Female (n=816)**	**Male (n=336)**	**Female (n=144)**	**Male (n=108)**
CRY2_rs11605924, CRY2_rs7945689	N = 50<br>Support = .061	N/A<br>Support < .05	N = 12<br>Support = .083	N = 6<br>Support = .056

Table S9 Association rule mining results 9

SNP set	Normal		Insulin resistance	
	**Female (n=816)**	**Male (n=336)**	**Female (n=144)**	**Male (n=108)**
TIMELESS_rs11171852, TIMELESS_rs774047	N = 74<br>Support = .091	N = 28<br>Support = .083	N = 11<br>Support = .076	N/A<br>Support < .05
TIMELESS_rs11171852, TIMELESS_rs774027	N = 74<br>Support = .091	N = 28<br>Support = .083	N = 11<br>Support = .076	N/A<br>Support < .05
TIMELESS_rs11171852, TIMELESS_rs11833583, TIMELESS_rs774047	N = 71<br>Support = .087	N = 28<br>Support = .083	N = 11<br>Support = .076	N/A<br>Support < .05
TIMELESS_rs11171852, TIMELESS_rs11833583, TIMELESS_rs774027	N = 71<br>Support = .087	N = 28<br>Support = .083	N = 11<br>Support = .076	N/A<br>Support < .05
TIMELESS_rs11171852, TIMELESS_rs11833583	N = 71<br>Support = .087	N = 28<br>Support = .083	N = 11<br>Support = .076	N/A<br>Support < .05
TIMELESS_rs11833583, TIMELESS_rs774027	N = 77<br>Support = .094	N = 33<br>Support = .098	N = 11<br>Support = .076	N/A<br>Support < .05
**PER3_rs17031578, PER3_rs228669**	**N = 42 *<br>Support = 0.051**	N = 21<br>Support = .063	**N = 15 * ‡<br>Support = .100**	N/A<br>Support < .05
TIMELESS_rs11171852, TIMELESS_rs774027, TIMELESS_rs774047	N = 74<br>Support = .091	N = 28<br>Support = .083	N = 11<br>Support = .076	N/A<br>Support < .05
TIMELESS_rs774027, TIMELESS_rs774047	N = 81<br>Support = .099	N = 33<br>Support = .098	N = 11<br>Support = .076	N/A<br>Support < .05
TIMELESS_rs11833583, TIMELESS_rs774027, TIMELESS_rs774047	N = 77<br>Support = .094	N = 33<br>Support = .098	N = 11<br>Support = .076	N/A<br>Support < .05
TIMELESS_rs11833583, TIMELESS_rs774047	N = 77<br>Support = .094	N = 33<br>Support = .098	N = 11<br>Support = .076	N/A<br>Support < .05

The difference within the same gender, significant at * p<.05, † p<.1
The difference between the different gender, significant at # p<.05, ‡ p<.1

Table S10 Association rule mining results 10

SNP set	Normal		Insulin resistance	
	**Female (n=816)**	**Male (n=336)**	**Female (n=144)**	**Male (n=108)**
LPL_rs291, LPL_rs301, LPL_rs320	N = 42<br>Support = .051	N = 17<br>Support = .051	N/A<br>Support < .05	N = 8<br>Support = .074
LPL_rs15285, LPL_rs295, LPL_rs301	N = 42<br>Support = .051	N = 17<br>Support = .051	N/A<br>Support < .05	N = 8<br>Support = .074
LPL_rs291, LPL_rs295, LPL_rs320	N = 42<br>Support = .051	N = 17<br>Support = .051	N/A<br>Support < .05	N = 8<br>Support = .074
LPL_rs291, LPL_rs326	N = 41<br>Support = .050	N = 17<br>Support = .051	N/A<br>Support < .05	N = 8<br>Support = .074
LPL_rs291, LPL_rs320	N = 42<br>Support = .051	N = 17<br>Support = .051	N/A<br>Support < .05	N = 8<br>Support = .074
LPL_rs295, LPL_rs301, LPL_rs326	N = 41<br>Support = .050	N = 18<br>Support = .054	N/A<br>Support < .05	N = 8<br>Support = .074

SIRT6_rs352492, SIRT6_rs352493	N = 64<br>Support = .078	N = 29<br>Support = .086	N/A<br>Support < .05	N = 9<br>Support = .083
LPL_rs295, LPL_rs301, LPL_rs320	N = 42<br>Support = .051	N = 17<br>Support = .051	N/A<br>Support < .05	N = 8<br>Support = .074
LPL_rs301, LPL_rs320	N = 42<br>Support = .051	N = 17<br>Support = .051	N/A<br>Support < .05	N = 8<br>Support = .074
LPL_rs15285, LPL_rs291, LPL_rs320	N = 42<br>Support = .051	N = 17<br>Support = .051	N/A<br>Support < .05	N = 8<br>Support = .074
LPL_rs295, LPL_rs320, LPL_rs326	N = 41<br>Support = .050	N = 17<br>Support = .051	N/A<br>Support < .05	N = 8<br>Support = .074
LPL_rs15285, LPL_rs295, LPL_rs320	N = 42<br>Support = .051	N = 17<br>Support = .051	N/A<br>Support < .05	N = 8<br>Support = .074
LPL_rs291, LPL_rs301	N = 42<br>Support = .051	N = 18<br>Support = .054	N/A<br>Support < .05	N = 8<br>Support = .074
LPL_rs295, LPL_rs301	N = 42<br>Support = .051	N = 18<br>Support = .054	N/A<br>Support < .05	N = 8<br>Support = .074
LPL_rs291, LPL_rs301, LPL_rs326	N = 41<br>Support = .050	N = 17<br>Support = .051	N/A<br>Support < .05	N = 8<br>Support = .074
SIRT6_rs352493, SIRT6_rs7260071	N = 64<br>Support = .078	N = 29<br>Support = .086	N/A<br>Support < .05	N = 9<br>Support = .083
LPL_rs295, LPL_rs320	N = 42<br>Support = .051	N = 17<br>Support = .051	N/A<br>Support < .05	N = 8<br>Support = .074
LPL_rs295, LPL_rs326	N = 41<br>Support = .050	N = 17<br>Support = .051	N/A<br>Support < .05	N = 8<br>Support = .074
LPL_rs301, LPL_rs320, LPL_rs326	N = 41<br>Support = .050	N = 17<br>Support = .051	N/A<br>Support < .05	N = 8<br>Support = .074
LPL_rs320, LPL_rs326	N = 43<br>Support = .053	N = 18<br>Support = .054	N/A<br>Support < .05	N = 8<br>Support = .074
SIRT6_rs352492, SIRT6_rs352493, SIRT6_rs7260071	N = 64<br>Support = .078	N = 29<br>Support = .086	N/A<br>Support < .05	N = 8<br>Support = .074
LPL_rs15285, LPL_rs291, LPL_rs301	N = 42<br>Support = .051	N = 17<br>Support = .051	N/A<br>Support < .05	N = 8<br>Support = .074
LPL_rs15285, LPL_rs301, LPL_rs320	N = 42<br>Support = .051	N = 17<br>Support = .051	N/A<br>Support < .05	N = 8<br>Support = .074
LPL_rs15285, LPL_rs291, LPL_rs295	N = 42<br>Support = .051	N = 17<br>Support = .051	N/A<br>Support < .05	N = 8<br>Support = .074
LPL_rs15285, LPL_rs320, LPL_rs326	N = 43<br>Support = .053	N = 18<br>Support = .054	N/A<br>Support < .05	N = 8<br>Support = .074
LPL_rs291, LPL_rs320, LPL_rs326	N = 41<br>Support = .050	N = 17<br>Support = .051	N/A<br>Support < .05	N = 8<br>Support = .074
LPL_rs291, LPL_rs295	N = 42<br>Support = .051	N = 18<br>Support = .054	N/A<br>Support < .05	N = 8<br>Support = .074
LPL_rs15285, LPL_rs301	N = 42<br>Support = .051	N = 17<br>Support = .051	N/A<br>Support < .05	N = 8<br>Support = .074
LPL_rs15285, LPL_rs301, LPL_rs326	N = 41<br>Support = .050	N = 17<br>Support = .051	N/A<br>Support < .05	N = 8<br>Support = .074
LPL_rs301, LPL_rs326	N = 41<br>Support = .050	N = 17<br>Support = .051	N/A<br>Support < .05	N = 8<br>Support = .074
LPL_rs15285, LPL_rs326	N = 43<br>Support = .053	N = 18<br>Support = .054	N/A<br>Support < .05	N = 8<br>Support = .074
LPL_rs15285, LPL_rs295, LPL_rs326	N = 41<br>Support = .050	N = 17<br>Support = .051	N/A<br>Support < .05	N = 8<br>Support = .074
LPL_rs15285, LPL_rs291	N = 42<br>Support = .051	N = 17<br>Support = .051	N/A<br>Support < .05	N = 8<br>Support = .074
LPL_rs15285, LPL_rs291, LPL_rs326	N = 41<br>Support = .050	N = 17<br>Support = .051	N/A<br>Support < .05	N = 8<br>Support = .074
LPL_rs291, LPL_rs295, LPL_rs301	N = 42<br>Support = .051	N = 18<br>Support = .054	N/A<br>Support < .05	N = 8<br>Support = .074
SIRT6_rs352492, SIRT6_rs7260071	N = 64<br>Support = .078	N = 29<br>Support = .086	N/A<br>Support < .05	N = 9<br>Support = .083
LPL_rs15285, LPL_rs320	N = 44<br>Support = .054	N = 18<br>Support = .054	N/A<br>Support < .05	N = 8<br>Support = .074

LPL_rs15285, LPL_rs295	N = 42<br>Support = .051	N = 17<br>Support = .051	N/A<br>Support < .05	N = 8<br>Support = .074
LPL_rs291, LPL_rs295, LPL_rs326	N = 41<br>Support = .050	N = 17<br>Support = .051	N/A<br>Support < .05	N = 8<br>Support = .074

Table S11 Association rule mining results 11

**SNP set**	**Normal**		**Insulin resistance**	
	**Female (n=816)**	**Male (n=336)**	**Female (n=144)**	**Male (n=108)**
PER2_rs4280405, PER2_rs934945	N = 56<br>Support = .069	N = 32<br>Support =.095	N = 12<br>Support = .083	N = 10<br>Support = .093
**NRF2_rs10930781, NRF2_rs2364720, NRF2_rs4243387**	**N = 47 †<br>Support = .058**	N = 24<br>Support = .071	**N = 14 †<br>Support = .097**	N = 11<br>Support = .100
NRF2_rs10188107, NRF2_rs2364720	N = 43<br>Support = .053	N = 23<br>Support = .068	N = 13<br>Support = .090	N = 11<br>Support = .100
**NRF2_rs10188107, NRF2_rs10930781, NRF2_rs4243387**	**N = 43 †<br>Support = .053**	N = 23<br>Support = .068	**N = 13 †<br>Support = .090**	N = 11<br>Support = .100
DEC1_rs10125253, DEC1_rs7850105	N = 65<br>Support = .080	N = 24<br>Support = .071	N = 9<br>Support = .063	N = 7<br>Support = .065
CLOCK_rs6832769, CLOCK_rs7673908	N = 47<br>Support = .058	N = 19<br>Support = .057	N = 10<br>Support = .069	N = 7<br>Support = .065
DEC1_rs10125253, DEC1_rs7026283	N = 65<br>Support = .080	N = 24<br>Support = .071	N = 9<br>Support = .063	N = 7<br>Support = .065
IRS1_rs16822642, IRS1_rs1801123	N = 44<br>Support = .054	N = 25<br>Support = .074	N = 9<br>Support = .063	N = 10<br>Support = .093
STAT3_rs1053005, STAT3_rs8064496	N = 55<br>Support = .067	N = 28<br>Support = .083	N = 10<br>Support = .069	N = 14<br>Support = .130
**NRF2_rs10188107, NRF2_rs2364720, NRF2_rs4243387**	**N = 43 †<br>Support = .053**	N = 23<br>Support = .068	**N = 13 †<br>Support = .090**	N = 11<br>Support = .100
DEC1_rs7026283, DEC1_rs7850105	N = 65<br>Support = .080	N = 24<br>Support = .071	N = 9<br>Support = .063	N = 7<br>Support = .065
DEC1_rs10125253, DEC1_rs7026283, DEC1_rs7850105	N = 65<br>Support = .080	N = 24<br>Support = .071	N = 9<br>Support = .063	N = 7<br>Support = .065
**NRF2_rs2364720, NRF2_rs4243387**	**N = 47 †<br>Support = .058**	N = 24<br>Support = .071	**N = 14 †<br>Support = .097**	N = 11<br>Support = .100
**NRF2_rs10188107, NRF2_rs10930781**	**N = 43 †<br>Support = .053**	N = 23<br>Support = .068	**N = 13 †<br>Support = .090**	N = 11<br>Support = .100
**NRF2_rs10188107, NRF2_rs4243387**	**N = 46 †<br>Support = .056**	N = 23<br>Support = .068	**N = 14 †<br>Support = .097**	N = 11<br>Support = .100
NRF2_rs10930781, NRF2_rs2364720	N = 60<br>Support = .074	N = 23<br>Support = .068	N = 15<br>Support = .100	N = 11<br>Support = .100
FOXO1_rs4325427, FOXO1_rs9532571	N = 59<br>Support = .072	N = 27 Support<br>= .080	N = 11<br>Support = .076	N = 8<br>Support = .074
**NRF2_rs10930781, NRF2_rs4243387**	**N = 47 †<br>Support = .078**	N = 24<br>Support = .071	**N = 14 †<br>Support = .097**	N = 11<br>Support = .100
**NRF2_rs10188107, NRF2_rs10930781, NRF2_rs2364720**	**N = 43 †<br>Support = .053**	N = 23<br>Support = .068	**N = 13 †<br>Support = .090**	N = 11<br>Support = .100

The difference within the same gender, significant at * p<.05, † p<.1
The difference between the different gender, significant at # p<.05, ‡ p<.1

Table S12 Common association patterns for normal and insulin-resistant males and females

Gene	SNP	Position	Minor allele	Major allele	MAF	Variant type	Publications (significance)
**SLC2A2**, Solute carrier family 2 member 2	rs28720688	chr3:171011340	G	A	G = 0.1902	intron variant	‣ N/A
	rs11924032	chr3:171017310	A	G	A = 0.1959	intron variant	‣ Dental caries, measured by decayed, missing, filled teeth (not significant, p=.36) [1]
	rs8192675	chr3:171007094	C	T	C = 0.2243	intron variant	‣ Higher prevalence of diabetes symptoms at diabetes diagnosis (C allele, p=.022 for polyuria, p=.033 for increased thirst) [2]<br>‣ .17% greater metformin-induced in hemoglobin A1c reduction (C allele, $p=6.6\times10^{-14}$) [3]<br>‣ Susceptibility to colorectal cancer (not significant, p=.75) [4]
	rs7356034	chr3:171014810	A	G	A = 0.1942	intron variant	‣ N/A
**RORA**, RAR related orphan receptor A	rs4775287	chr15:60695031	C	T	C = 0.2877	intron variant	‣ N/A
	rs999449	chr15:60696792	A	C	A = 0.3074	intron variant	‣ N/A
	rs2414682	chr15:60894314	C	T	C = 0.1926	intron variant	‣ N/A
	rs4774376	chr15:60896482	G	C	G = 0.2815	intron variant	‣ N/A
	rs7177996	chr15:60865889	T	C	T = 0.2858	intron variant	‣ N/A
	rs7182392	chr15:60880331	T	C	T = 0.2354	intron variant	‣ Cognitive aging, measured by Mini-Mental State Examinations (not significant after Bonferroni correction, p=.025) [5]
**NPAS2**, Neuronal PAS domain protein 2	rs3754675	chr2:100933381	C	T	C = 0.2934	intron variant	‣ Late-onset Alzheimer's disease (T allele, p=.014) [6]
	rs72627425	chr2:100887963	A	G	A = 0.2984	intron variant	‣ N/A
	rs17020663	chr2:100961588	C	G	C = 0.2256	intron variant	‣ N/A
	rs57069400	chr2:100960284	T	C	T = 0.2238	intron variant	‣ N/A
**IRS1**, Insulin receptor substrate 1	rs13431179	chr2:226745525	A	G	A = 0.3037	intron variant	‣ Cardiovascular clinical events among African Americans (local European ancestry region from rs13431179(2:227.318) to rs3731597(2:227.371), $p=6.7\times10^{-4}$) [7]
	rs1560252	chr2:226788086	C	T	C = 0.2213	intron variant	‣ N/A
	rs1801123	chr2:226796327	C	T	C = 0.2655	synonymous variant; coding sequence variant	‣ Colorectal cancer risk (TC and TT genotype, combined with IRS rs1801278 CC, AKT2 rs3730256 AA, and AKT2 rs7247515 TT genotypes) [8]

							‣ Worse overall survival among metastatic colorectal cancer (C allele, p=.029) [9]<br>‣ Autism spectrum disorder (A allele, p=.022) [10]<br>‣ Breast and ovarian cancer risk for BRCA1 or BRCA2 mutant carriers (not significant) [11]<br>‣ Type 1 diabetes (not significant) [12]<br>‣ Breast cancer risk (G allele, unadjusted p=.017) [13]
	rs10205233	chr2:226749289	T	C	T = 0.2023	intron variant	‣ Gastric cancer risk (CT genotype, p=.031) [14]
	rs16822574	chr2:226737565	T	C	T = 0.2223	intron variant	‣ N/A
	rs13431554	Chr2:226732872	G	A	G = 0.2219	3'-UTR variant	‣ High platelet reactivity with clopidogrel therapy in coronary artery disease patients with type 2 diabetes (G allele, p<.001) [15]
	rs16822642	chr2:226784837	T	C	T = 0.2623	intron variant	‣ N/A
**LPL**, Lipoprotein lipase	rs320	chr8:19961566	G	T	G = 0.2288	intron variant	‣ Coronary artery disease (not significant, p=.35) [16], (not significant, p=.087) [17]<br>‣ Higher levels of triglyceride (T allele, p=.0028), leptin (T allele, p=.0010) [18]<br>‣ Obesity (not significant, p=.26), protective effect on hypertriglyceridemia in women (TT genotype, p=.037) [19]<br>‣ Lower high-density lipoprotein-cholesterol level (rs320≠TT or rs708272≠TT, p=.011) [20]<br>‣ Dyslipidemia (G allele, p<.001) [21]<br>‣ Myocardial infarction (T allele, p=.032) [22]<br>‣ Ischemic stroke (not significant, p=.819) [23], (not significant, p=.73) [24]<br>‣ Diabetic dyslipidemia (G allele, p<.001) [25]<br>‣ Low-density lipoprotein-cholesterol lowering effect of atorvastatin in ischemic stroke patients (T allele, p=.008) [26]<br>‣ Acute non-biliary pancreatitis (T allele, p=.03) [27]<br>‣ Postprandial triacylglycerol and glucose response to sequential meal ingestion (not significant) [28]<br>‣ Lower triglyceride and high-density lipoprotein-cholesterol (T allele, p<.001 and p=.02, respectively) [29]<br>‣ Differences in triglyceride (not significant), low-density lipoprotein-cholesterol (p=.029), high-density lipoprotein-cholesterol (p=.018), apoprotein B (p=.031) and apolipoprotein A1 (not significant) levels [30]<br>‣ Interaction with dietary polyunsaturated fatty acids for body mass index and waist circumference (p=.002 and p=.001, respectively) [31]<br>‣ Triglyceride lowering effect (G allele, p<.0001) [32]<br>‣ Higher high-density lipoprotein-cholesterol and lower triglyceride levels (GG genotype, not significant) [33]
	rs301	chr8:19959423	C	T	C = 0.224	intron variant	‣ Differences in triglyceride (not significant), low-density lipoprotein-cholesterol (not significant), high-density lipoprotein-cholesterol

							(p=.008), apoprotein B (not significant) and apolipoprotein A1 (p=.035) levels [30]<br>‣ Vascular dysfunction (p=.025), elevated plasma glucose ($p=8.0\times10^{-3}$), atherogenetic dyslipidemia ($p=1.4\times10^{-8}$), vascular inflammation (not significant), pro-thrombotic state (not significant), central obesity (p=.035) [34]<br>‣ Lower triglyceride (C allele, p<.0001) [35]<br>‣ Differences in high-density lipoproteon-cholesterol and apoprotein A1 levels ($p=9.0\times10^{-11}$, and $p\text{-}4\times10^{-6}$, respectively) [36]<br>‣ Differences in high-density lipoprotein-cholesterol level ($p=8.8\times10^{-3}$) [37]<br>‣ Differences in high-density lipoprotein-cholesterol level and waist circumference ($p=3.2\times10^{-11}$) [38]
	rs326	chr8:19961928	G	A	G = 0.2248	intron variant	‣ Body mass index and its response to diet intervention (not significant) [39]<br>‣ Lower triglyceride level (G allele, $p=2.3\times10^{-6}$), higher high-density lipoprotein-cholesterol level (G allele, $p=9.7\times10^{-4}$) [40]<br>‣ Differences in total cholesterol (not significant), lower triglyceride (G allele, p<.005), higher high-density lipoprotein-cholesterol (G allele, p<.008), and higher low-density lipoprotein-cholesterol (G allele, not significant) levels [41]
	rs291	chr8:19958341	C	T	C = 0.2208	intron variant	‣ N/A
	rs295	chr8:19958727	C	A	C = 0.2216	intron variant	‣ Differences in triglyceride (not significant), low-density lipoprotein-cholesterol (not significant), high-density lipoprotein-cholesterol (p=.043), apolipoprotein B (not significant), and apolipoprotein A1 (not significant) levels [30]<br>‣ Higher high-density lipoprotein-cholesterol (C allele, p=.002), and lower triglyceride (C allele, $p=7.5\times10^{-4}$) levels [42]<br>‣ Metabolic syndrome ($p=1.7\times10^{-9}$) [38]<br>‣ Differences in triglyceride ($p=3.4\times10^{-3}$), and high-density lipoprotein cholesterol ($p=8.3\times10^{-3}$) levels [37]
	rs13702	chr8:19966981	C	T	C = 0.2253	3'-UTR variant	‣ Lower mean high-density lipoprotein-cholesterol concentrations when consuming total fat and monounsaturated fat above the sample medians (G allele, p<.05) [43]<br>‣ Lower visceral fat (CC genotype, p=.017) and android fat mass (CC genotype, p=.037) following average consumption of monounsaturated fatty acid diets [44]<br>‣ Differences in total cholesterol, high-density lipoprotein-cholesterol, low-density lipoprotein-cholesterol, triglyceride, and very-low-density lipoprotein-cholesterol levels (not significant) [45]<br>‣ Type 2 diabetes (CT genotype, p=.005, CC genotype, p=.010) [46]<br>‣ Nonalcoholic fatty liver disease (not significant) [47]<br>‣ Higher triglyceride level (GG genotype, p=.004) [48]

							‣ Differences in triglyceride (not significant), low-density lipoprotein-cholesterol (not significant), high-density lipoprotein-cholesterol (p=.011), apolipoprotein B (not significant), and apolipoprotein A1 (not significant) levels [30]<br>‣ Difference in high-density lipoprotein-cholesterol level ($p=3.44\times10^{-6}$) [49]<br>‣ Differences in body mass index (not significant), waist circumference (not significant), no significant interactions with saturated or monounsaturated fatty acids [31]<br>‣ Association with high-density lipoprotein cholesterol ($p=1.34\times10^{-9}$) [50]<br>‣ Association with high-density lipoprotein cholesterol (in European, $p=2.14\times10^{-28}$, in African American, $p=3.12\times10^{-11}$) [51]<br>‣ Differences in high-density lipoprotein cholesterol and triglyceride levels ($p=1.0\times10^{-16}$) [38]<br>‣ Association with triglyceride ($p=6.9\times10^{-5}$) [52], ($p=1.7\times10^{-6}$) [53], ($p=1.1\times10^{-3}$) [37]<br>‣ Association with high-density lipoprotein cholesterol (in males, p=.009, in females, p=.006) [54]
	rs15285	chr8:19967156	T	C	T = 0.2249	3'-UTR variant	‣ Differences in triglyceride ($p=5.9\times10^{-3}$), and high-density lipoprotein cholesterol ($p=6.9\times10^{-4}$) levels [37]<br>‣ Differences in triglyceride and blood pressure ($p=1.3\times10^{-10}$) [38]
**CRY2**, Cryptochrome 2	rs11605924	chr11:45851540	C	A	C = 0.2313	Intron Variant	‣ Increased gestational diabetes mellitus risk in Scandinavian (p=.014) but decreased gestational diabetes mellitus risk in Punjabi Indian women (p=.002) [55]<br>‣ Reduced risk of gestational diabetes mellitus in Punjabi Indian women (p=.038) [56]<br>‣ Reduced risk of type 2 diabetes mellitus in the Saudi Arabian population ($p=4.9\times10^{-3}$) [57]<br>‣ Fasting plasma glucose levels in Mexican children and adolescents (not significant, $p=1.1\times10^{-2}$) [58]<br>‣ Type 2 Diabetes risk in the Qatari population (not significant, p=.50) [59]<br>‣ Fasting glycemia (p<.0005) [60]<br>‣ Interaction with sleep duration, resulting in differences in high-density lipoprotein cholesterol (0.010 mmol/L higher HDL-c with each additional hour of sleep in the presence of A allele, p<.01) [61]<br>‣ differences in fasting (p<.0001) and 2 hours (p=.04) glucose concentrations in the GLACIER cohort [62]<br>‣ Greater reduction in respiratory quotient (p=.03) greater increase in resting metabolic rate (A allele, p=.04) [63]<br>‣ Risk of incident cardiovascular diseases (not significant) [64]<br>‣ Differences in fasting glucose, insulin, and proinsulin levels, insulinogenic index, insulin sensitivity index, oral disposition index (not significant) [65]<br>‣ Type 2 diabetes risk in South Asians (not significant) [66]

							‣ Fasting glucose levels (not significant) [67]<br>‣ Increased risk of combined impaired fasting glucose and type 2 diabetes (A allele, p=.04) [68]<br>‣ Differences in fasting glucose levels (p=.015) [69]<br>‣ Differences in fasting glucose levels in African Americans (not significant) [70]<br>‣ Type 2 diabetes (p=.049, not significant when adjusted) [71]<br>‣ Higher fasting glucose levels (A allele, p=1.5×10^{-6}), 2-hour glucose levels (A allele, p=2.0×10^{-4}) [72]
	rs7945689	chr11:45857455	T	C	T = 0.2311	Intron Variant	‣ N/A
PER2	rs4280405	chr2:238249900	T	C	T = 0.29	Intron Variant	‣ N/A
	rs934945	chr2:238246412	T	C	T = 0.2897	Missense Variant	‣ Associations between the composite scale of morningness subscale scores of morning alertness (p=.026) and activity planning(p=.0031) [73]<br>‣ Differences in the composite scale for morningness score (p=.031), morning alertness score (not significant), activity planning score (p=.003), and the total score (p=.012) [74]<br>‣ Increased risk of estrogen-/progesteron-positive tumors in breast cancer (dominant genetic model, p=.01) [75]<br>‣ Association with cluster headache (not significant) [76]<br>‣ Susceptibility to systemic lupus erythematosus (not significant) [77]<br>‣ Differences in age at onset of psychosis (p=.012), clinical global impression score for psychosis (p=.033) [78]<br>‣ Rheumatoid arthritis (not significant) [79]<br>‣ Decreased odds of fatigue in malignant glioma patients (T allele, p<.05) [80]<br>‣ Daytime sleepiness in myocardial infarction patients (p=.044) [81]<br>‣ Reduced predisposition to sarcoma (T allele, p=.02) [82]<br>‣ Colorectal cancer susceptibility (not significant) [83]<br>‣ Plasma saturated fatty acid levels in metabolic syndrome patients (not significant) [84]<br>‣ Composite scale of morningness score, total score (p=.010), morningness score (p=.018), and activity planning score (p=.005) in Korean [85]<br>‣ Noise-induced hearing loss (not significant) [86]
**NRF2**, Nuclear factor erythroid-2-related factor 2	rs10930781	chr2:177249904	A	G	A = 0.2783	Intron Variant	‣ CC genotype at rs1093781 and GG genotype at rs1048290 scored higher on IQ test compared to T carrier-C carrier genotype or CC-C carrier genotype (p>.05) [87]<br>‣ Acute respiratory distress syndrome susceptibility (p=.0085) [88]
	rs2364720	chr2:177240416	A	G	A = 0.284	Intron Variant	‣ Acute respiratory distress syndrome susceptibility (p=.0085) [88]
	rs10188107	chr2:177255584	T	G	T = 0.2549	Intron Variant	‣ Acute respiratory distress syndrome susceptibility (p=.0071) [88]

	rs4243387	chr2:177253037	C	T	C = 0.2777	Intron Variant	‣ Increased risk of anti-tuberculosis drug-induced liver injury (TC genotype, p=.038) [89]<br>‣ Acute respiratory distress syndrome susceptibility (p=.0071) [88]<br>‣ All-cause, cardiovascular, and chronic obstructive pulmonary disease mortality risk (not significant) [90]<br>‣ Differences in forced expiratory volume in 1 second in the general population (not significant) [91]
**DEC1**, Deleted in esophageal cancer 1	rs7850105	chr9:115257508	C	T	C = 0.278	Intron Variant	‣ N/A
	rs10125253	chr9:115250562	T	C	T = 0.2788	Intron Variant	‣ N/A
	rs7026283	chr9:115258346	G	C	G = 0.2783	Intron Variant	‣ N/A
	rs10817745	chr9:115323089	A	G	A = 0.2827	Intron Variant	‣ N/A
	rs10982725	chr9:115399442	A	G	A = 0.2803	Intron Variant	‣ N/A
**CLOCK**, Circadian locomotor output cycles kaput	rs6832769	chr4:55432027	G	A	G = 0.2623	Intron Variant	‣ Association with agreeableness in the five-factor model of personality ($p=9\times10^{-6}$) [92]<br>‣ Higher emotional prosocial tendencies measure score (A allele, p=.028) [93]
	rs7673908	chr4:55528652	G	A	G = 0.2634	Intron Variant	‣ N/A
**STAT3**, Signal transducer and activator of transcription 3	rs1053005	chr17:42313892	C	T	C = 0.3011	3’-UTR Variant	‣ Susceptibility to tuberculosis (AA genotype, p=.036) [94]<br>‣ Susceptibility to tuberculosis in western Chinese Han population (not significant) [95]<br>‣ Decreased susceptibility to epilepsy (GG genotype, p<.05) [96]<br>‣ Susceptibility to rheumatoid arthritis in the Polish population (not significant) [97]<br>‣ Susceptibility to chronic hepatitis B virus infection (GG genotype, p=.046) [98]<br>‣ Gastric cancer risk in eastern Chinse population (not significant) [99]<br>‣ Decreased susceptibility to Graves' disease and Hashimoto's disease (A allele, p<.01) [100]<br>‣ Colon and rectal cancer risk (not significant) [101]<br>‣ Prostate cancer risk (not significant) [102]<br>‣ Ankylosing spondylitis risk in Han Chinese (p=.017) [103]<br>‣ Abdominal obesity risk (G allele, p=.0033) [104]
	rs8064496	chr17:42322846	G	A	G = 0.2944	Intron Variant	‣ N/A
**FOXO1**, Forkhead box protein O1	rs4325427	chr13:40642397	C	T	C = 0.2721	Intron Variant	‣ N/A
	rs9532571	chr13:40644533	C	T	C = 0.2759	Intron Variant	‣ Bacteremia risk (not significant) [105]
	rs17031578	chr1:7799131	C	A	C = 0.2438	Intron Variant	‣ N/A

**PER3**, Period circadian regulator 3	rs228669	chr1:7809988	T	C	T = 0.2434	Synonymous Variant	‣ Gene-gene interactions among NR1D1 rs2314339, TIMELESS rs4630333, and PER3 rs228669 were significantly associated with chronotype (p<.001) [106]<br>‣ Marginal association with preterm birth (p=.02) [107]<br>‣ Overall survival and response to chemotherapy in gastric cancer patients (not significant) [108]<br>‣ Overall survival in hepatocellular carcinoma patients (p=.03) [109]
**TIMELESS**, timeless circadian regulator	rs11171852	chr12:56439440	T	C	T = 0.2869	Intron Variant	‣ N/A
	rs774047	chr12:56422138	C	T	C = 0.2963	Missense Variant	‣ Susceptibility to breast cancer (not significant) [110]<br>‣ Morning, intermediate, evening, delayed sleep, and free-running type (not significant) [111]
	rs774027	chr12:56428594	T	A	T = 0.2952	Missense Variant	‣ N/A
	rs11833583	chr12:56435086	T	G	T = 0.2943	Intron Variant	‣ N/A
**BMAL1**, brain and muscle ARNT-like 1	rs57069400	chr2:100960284	T	C	T = 0.2238	Intron Variant	‣ N/A
	rs11022759	chr11:13305228	A	G	A = 0.2257	Intron Variant	‣ N/A
**SIRT6**, Sirtuin 6	rs352492	chr19:4179639	T	C	T = 0.2832	Intron Variant	‣ N/A
	rs7260071	chr19:4180693	T	G	T = 0.3026	Synonymous Variant	‣ N/A
	rs352493	chr19:4180839	C	T	C = 0.2842	Missense Variant	‣ Severity of coronary artery disease in Chinese Han population (C allele, adjusted p=.013) [112]<br>‣ Longevity in the Chinese population (not significant) [113]
JAK2	rs4495487	chr9:5072798	C	T	C = 0.2304	Intron Variant	‣ Polycythemia vera risk (T allele, p<.0001), myeloproliferative neoplasm predisposition (C allele) in the Japanese population [114]
	rs3780381	chr9:5114523	C	A	C = 0.2297	Intron Variant	‣ Colon and rectal cancer risk (not significant) [101]<br>‣ Polycythemia vera risk ($p=3.6\times10^{-6}$) [115]
	rs3824433	chr9:5113577	T	C	T = 0.2343	Intron Variant	‣ Placental malaria infection risk ($p=7.0\times10^{-4}$) [116]

[1] Robino A, Bevilacqua L, Pirastu N, Situlin R, Di Lenarda R, Gasparini P, Navarra CO. Polymorphisms in sweet taste genes (TAS1R2 and GLUT2), sweet liking, and dental caries prevalence in an adult Italian population. Genes & nutrition. 2015 Sep;10(5):1-9.

[2] Rathmann W, Strassburger K, Bongaerts B, Kuss O, Müssig K, Burkart V, Szendroedi J, Kotzka J, Knebel B, Al-Hasani H, Roden M. A variant of the glucose transporter gene SLC2A2 modifies the glycaemic response to metformin therapy in recently diagnosed type 2 diabetes. Diabetologia. 2019 Feb 1;62(2):286-91.

[3] Zhou K, Yee SW, Seiser EL, Van Leeuwen N, Tavendale R, Bennett AJ, Groves CJ, Coleman RL, Van Der Heijden AA, Beulens JW, De Keyser CE. Variation in the glucose transporter gene SLC2A2 is associated with glycemic response to metformin. Nature genetics. 2016 Sep;48(9):1055-9.

[4] Huhn S, Bevier M, Rudolph A, Pardini B, Naccarati A, Hein R, Hoffmeister M, Vodickova L, Novotny J, Brenner H, Chang-Claude J. Shared ancestral susceptibility to colorectal cancer and other

nutrition related diseases. BMC medical genetics. 2012 Dec;13(1):1-2.

[5] Lin, E., Kuo, P. H., Liu, Y. L., Yang, A. C., Kao, C. F., & Tsai, S. J. (2017). Effects of circadian clock genes and environmental factors on cognitive aging in old adults in a Taiwanese population. *Oncotarget*, *8*(15), 24088.

[6] Abraham R, Moskvina V, Sims R, Hollingworth P, Morgan A, Georgieva L, Dowzell K, Cichon S, Hillmer AM, O'Donovan MC, Williams J. A genome-wide association study for late-onset Alzheimer's disease using DNA pooling. BMC medical genomics. 2008 Dec;1(1):1-3.

[7] Shendre A, Irvin MR, Wiener H, Zhi D, Limdi NA, Overton ET, Shrestha S. Local ancestry and clinical cardiovascular events among African Americans from the atherosclerosis risk in communities study. Journal of the American Heart Association. 2017 Apr 10;6(4):e004739.

[8] Jung SY, Zhang ZF. The effects of genetic variants related to insulin metabolism pathways and the interactions with lifestyles on colorectal cancer risk. Menopause (New York, NY). 2019 Jul;26(7):771.

[9] Schirripa M, Zhang W, Heinemann V, Cao S, Okazaki S, Yang D, Loupakis F, Berger MD, Ning Y, Miyamoto Y, Suenaga M. Single nucleotide polymorphisms in the IGF-IRS pathway are associated with outcome in mCRC patients enrolled in the FIRE-3 trial. International journal of cancer. 2017 Jul 15;141(2):383-92.

[10] Park HJ, Kim SK, Kang WS, Park JK, Kim YJ, Nam M, Kim JW, Chung JH. Association between IRS1 gene polymorphism and autism spectrum disorder: a pilot case-control study in Korean males. International journal of molecular sciences. 2016 Aug;17(8):1227.

[11] Ding YC, McGuffog L, Healey S, Friedman E, Laitman Y, Kaufman B, Liljegren A, Lindblom A, Olsson H, Kristoffersson U, Stenmark-Askmalm M. A nonsynonymous polymorphism in IRS1 modifies risk of developing breast and ovarian cancers in BRCA1 and ovarian cancer in BRCA2 mutation carriers. Cancer Epidemiology and Prevention Biomarkers. 2012 Aug 1;21(8):1362-70.

[12] Bergholdt R, Brorsson C, Boehm B, Morahan G, Pociot F. No association of the IRS1 and PAX4 genes with type I diabetes. Genes & Immunity. 2009 Dec;10(1):S49-53.

[13] Neuhausen SL, Brummel S, Ding YC, Singer CF, Pfeiler G, Lynch HT, Nathanson KL, Rebbeck TR, Garber JE, Couch F, Weitzel J. Genetic variation in insulin-like growth factor signaling genes and breast cancer risk among BRCA1 and BRCA2 carriers. Breast Cancer Research. 2009 Oct;11(5):1-2.

[14] Zheng W, Wu C, Wu X, Cai Y, Liu B, Wang C. Genetic variants of autophagy-related genes in the PI3K/Akt/mTOR pathway and risk of gastric cancer in the Chinese population. Gene. 2021 Feb 15;769:145190.

[15] Zhang D, Zhang X, Liu D, Liu T, Cai W, Yan C, Han Y. Association between insulin receptor substrate-1 polymorphisms and high platelet reactivity with clopidogrel therapy in coronary artery disease patients with type 2 diabetes mellitus. Cardiovascular diabetology. 2016 Dec;15(1):1-8.

[16] Bogari NM, Allam RM, Bouazzaoui A, Elkhateeb O, Porqueddu M, Colombo GI. Coronary Artery Disease: Association Study of 5 Loci with Angiographic Indices of Disease Severity. Disease markers. 2021 Jul 12;2021.

[17] Bogari NM, Aljohani A, Dannoun A, Elkhateeb O, Porqueddu M, Amin AA, Bogari DN, Taher MM, Buba F, Allam RM, Bogari MN. Association between HindIII (rs320) variant in the lipoprotein lipase gene and the presence of coronary artery disease and stroke among the Saudi population. Saudi Journal of Biological Sciences. 2020 Aug 1;27(8):2018-24.

[18] Peacock RE, Hamsten A, Nilsson-Ehle P, Humphries SE. Associations between lipoprotein lipase gene polymorphisms and plasma correlations of lipids, lipoproteins and lipase activities in young myocardial infarction survivors and age-matched healthy individuals from Sweden. Atherosclerosis. 1992 Dec 1;97(2-3):171-85.

[19] Alinaghian N, Abdollahi E, Torab M, Khodaparast M, Zamani F, Rahimi-Moghaddam P. Gender-related relation between metabolic syndrome and S447X and HindIII polymorphisms of lipoprotein lipase gene in northern Iran. Gene. 2019 Jul 20;706:13-8.

[20] Moghadasi M, Kelishadi R, Marateb HR, Javanmard SH, Mansourian M, Heshmat R, Motlagh ME. Logic regression analysis of gene polymorphisms and HDL levels in a nationally representative sample of Iranian adolescents: The CASPIAN-III study. International journal of endocrinology and metabolism. 2017 Jul;15(3).

[21] Marateb HR, Mohebian MR, Javanmard SH, Tavallaei AA, Tajadini MH, Heidari-Beni M, Mañanas MA, Motlagh ME, Heshmat R, Mansourian M, Kelishadi R. Prediction of dyslipidemia using gene mutations, family history of diseases and anthropometric indicators in children and adolescents: the CASPIAN-III study. Computational and structural biotechnology journal. 2018 Jan 1;16:121-30.

[22] Kukava NG, Titov BV, Osmak GJ, Matveeva NA, Kulakova OG, Favorov AV, Shakhnovich RM, Ruda MY, Favorova OO. Multilocus analysis of genetic susceptibility to myocardial infarction in Russians: replication study. Acta Naturae (англоязычная версия). 2017;9(4 (34)).

[23] Yue YH, Liu LY, Hu L, Li YM, Mao JP, Yang XY, Dila NM. The association of lipid metabolism relative gene polymorphisms and ischemic stroke in Han and Uighur population of Xinjiang. Lipids in health and disease. 2017 Dec;16(1):1-8.

[24] Velásquez Pereira LC, Vargas Castellanos CI, Silva Sieger FA. Polymorphisms of the lipoprotein lipase gene as genetic markers for stroke in colombian population: a case control study. Colombia Médica. 2016 Dec;47(4):189-95.

[25] Vardarlı AT, Harman E, Çetintaş VB, Kayıkçıoğlu M, Vardarlı E, Zengi A, Küçükaslan AŞ, Eroğlu Z. Polymorphisms of lipid metabolism enzyme-coding genes in patients with diabetic dyslipidemia. Anatolian journal of cardiology. 2017 Apr;17(4):313.

[26] Yue YH, Bai XD, Zhang HJ, Li YM, Hu L, Liu LY, Mao JP, Yang XY, Dila NM. Gene polymorphisms affect the effectiveness of atorvastatin in treating ischemic stroke patients. Cellular Physiology and Biochemistry. 2016;39(2):630-8.

[27] Samgina TA, Bushueva OY, Nazarenko PM, Polonikov AV. Association of the HindIII lipoprotein lipase gene polymorphism with the development of the non-biliary acute pancreatitis: a pilot study. Bulletin of experimental biology and medicine. 2016 May;161(1):79-82.

[28] Shatwan IM, Minihane AM, Williams CM, Lovegrove JA, Jackson KG, Vimaleswaran KS. Impact of lipoprotein lipase gene polymorphism, S447X, on postprandial triacylglycerol and glucose response to sequential meal ingestion. International journal of molecular sciences. 2016 Mar;17(3):397.

[29] Askari G, Heidari-Beni M, Mansourian M, Esmaeil-Motlagh M, Kelishadi R. Interaction of lipoprotein lipase polymorphisms with body mass index and birth weight to modulate lipid profiles in children and adolescents: the CASPIAN-III Study. Sao Paulo Medical Journal. 2016 Jan 19;134:121-9.

[30] Pirim D, Wang X, Radwan ZH, Niemsiri V, Bunker CH, Barmada MM, Kamboh MI, Demirci FY. Resequencing of LPL in African Blacks and associations with lipoprotein–lipid levels. European Journal of Human Genetics. 2015 Sep;23(9):1244-53.

[31] Ma Y, Tucker KL, Smith CE, Lee YC, Huang T, Richardson K, Parnell LD, Lai CQ, Young KL, Justice AE, Shao Y. Lipoprotein lipase variants interact with polyunsaturated fatty acids for obesity traits in women: replication in two populations. Nutrition, Metabolism and Cardiovascular Diseases. 2014 Dec 1;24(12):1323-9.

[32] Ariza MJ, Sánchez-Chaparro MÁ, Barón FJ, Hornos AM, Calvo-Bonacho E, Rioja J, Valdivielso P, Gelpi JA, González-Santos P. Additive effects of LPL, APOA5 and APOE variant combinations on triglyceride levels and hypertriglyceridemia: results of the ICARIA genetic sub-study. BMC medical genetics. 2010 Dec;11(1):1-0.

[33] Javorský M, Gašperíková D, Ukropec J, Sedláková B, Riečanský I, Križanová OG, Šeböková E, Dobríková M, Klimeš I, Tkáč I. Lipoprotein lipase Hin dIII polymorphism influences HDL-cholesterol levels in statin-treated patients with coronary artery disease. Wiener klinische Wochenschrift. 2007 Aug;119(15):476-82.

[34] Avery CL, He Q, North KE, Ambite JL, Boerwinkle E, Fornage M, Hindorff LA, Kooperberg C, Meigs JB, Pankow JS, Pendergrass SA. A phenomics-based strategy identifies loci on APOC1, BRAP, and PLCG1 associated with metabolic syndrome phenotype domains. PLoS genetics. 2011 Oct 13;7(10):e1002322.

[35] Smith AJ, Palmen J, Putt W, Talmud PJ, Humphries SE, Drenos F. Application of statistical and functional methodologies for the investigation of genetic determinants of coronary heart disease biomarkers: lipoprotein lipase genotype and plasma triglycerides as an exemplar. Human molecular genetics. 2010 Oct 15;19(20):3936-47.

[36] Talmud PJ, Drenos F, Shah S, Shah T, Palmen J, Verzilli C, Gaunt TR, Pallas J, Lovering R, Li K, Casas JP. Gene-centric association signals for lipids and apolipoproteins identified via the HumanCVD BeadChip. The American journal of human genetics. 2009 Nov 13;85(5):628-42.

[37] Deo RC, Reich D, Tandon A, Akylbekova E, Patterson N, Waliszewska A, Kathiresan S, Sarpong D, Taylor Jr HA, Wilson JG. Genetic differences between the determinants of lipid profile phenotypes in African and European Americans: the Jackson Heart Study. PLoS genetics. 2009 Jan 16;5(1):e1000342.

[38] Kraja AT, Vaidya D, Pankow JS, Goodarzi MO, Assimes TL, Kullo IJ, Sovio U, Mathias RA, Sun YV, Franceschini N, Absher D. A bivariate genome-wide approach to metabolic syndrome: STAMPEED consortium. Diabetes. 2011 Apr 1;60(4):1329-39.

[39] Franzago M, Di Nicola M, Fraticelli F, Marchioni M, Stuppia L, Vitacolonna E. Nutrigenetic variants and response to diet/lifestyle intervention in obese subjects: a pilot study. Acta Diabetologica. 2021 Sep 3:1-3.

[40] Liu Y, Zhou D, Zhang Z, Song Y, Zhang D, Zhao T, Chen Z, Sun Y, Zhang D, Yang Y, Xing Q. Effects of genetic variants on lipid parameters and dyslipidemia in a Chinese population [S]. Journal of lipid research. 2011 Feb 1;52(2):354-60.

[41] Tang W, Apostol G, Schreiner PJ, Jacobs Jr DR, Boerwinkle E, Fornage M. Associations of lipoprotein lipase gene polymorphisms with longitudinal plasma lipid trends in young adults: The Coronary Artery Risk Development in Young Adults (CARDIA) study. Circulation: Cardiovascular Genetics. 2010 Apr;3(2):179-86.

[42] Pirim D, Wang X, Radwan ZH, Niemsiri V, Hokanson JE, Hamman RF, Barmada MM, Demirci FY, Kamboh MI. Lipoprotein lipase gene sequencing and plasma lipid profile. Journal of lipid research. 2014 Jan 1;55(1):85-93.

[43] Hannon BA, Edwards CG, Thompson SV, Burke SK, Burd NA, Holscher HD, Teran-Garcia M, Khan NA. Genetic Variants in Lipid Metabolism Pathways Interact with Diet to Influence Blood Lipid Concentrations in Adults with Overweight and Obesity. Lifestyle Genomics. 2020;13(6):155-63.

[44] Hammad SS, Eck P, Sihag J, Chen X, Connelly PW, Lamarche B, Couture P, Guay V, Maltais-Giguère J, West SG, Kris-Etherton PM. Common Variants in Lipid Metabolism–Related Genes Associate with Fat Mass Changes in Response to Dietary Monounsaturated Fatty Acids in Adults with Abdominal Obesity. The Journal of nutrition. 2019 Oct 1;149(10):1749-56.

[45] Al-Bustan SA, Al-Serri A, Alnaqeeb MA, Annice BG, Mojiminiyi O. Genetic association of LPL rs1121923 and rs258 with plasma TG and VLDL levels. Scientific reports. 2019 Apr 3;9(1):1-0.

[46] Hatefi Z, Soltani G, Khosravi S, Kazemi M, Salehi AR, Salehi R. Micro R-410 binding site single nucleotide polymorphism rs13702 in lipoprotein lipase gene is effective to increase susceptibility to type 2 diabetes in iranian population. Advanced biomedical research. 2018;7.

[47] Yang H, Chen G, Song C, Li D, Ma Q, Chen G, Li X. A novel index including SNPs for the screening of nonalcoholic fatty liver disease among elder Chinese: A population-based study. Medicine. 2018 Mar;97(13).

[48] Wu S, Hsu LA, Teng MS, Lin JF, Chou HH, Lee MC, Wu YM, Su CW, Ko YL. Interactive effects of C-reactive protein levels on the association between APOE variants and triglyceride levels in a Taiwanese population. Lipids in health and disease. 2016 Dec;15(1):1-1.

[49] Shetty PB, Tang H, Feng T, Tayo B, Morrison AC, Kardia SL, Hanis CL, Arnett DK, Hunt SC, Boerwinkle E, Candidate Gene Association Resource (CARe) Consortium. Variants for HDL-C, LDL-C, and triglycerides identified from admixture mapping and fine-mapping analysis in African American families. Circulation: Cardiovascular Genetics. 2015 Feb;8(1):106-13.

[50] Elbers CC, Guo Y, Tragante V, Van Iperen EP, Lanktree MB, Castillo BA, Chen F, Yanek LR, Wojczynski MK, Li YR, Ferwerda B. Gene-centric meta-analysis of lipid traits in African, East Asian

and Hispanic populations. PloS one. 2012 Dec 7;7(12):e50198.

[51] Musunuru K, Romaine SP, Lettre G, Wilson JG, Volcik KA, Tsai MY, Taylor Jr HA, Schreiner PJ, Rotter JI, Rich SS, Redline S. Multi-ethnic analysis of lipid-associated loci: the NHLBI CARe project. PloS one. 2012 May 21;7(5):e36473.

[52] Voruganti VS, Cole SA, Ebbesson SO, Göring HH, Haack K, Laston S, Wenger CR, Tejero ME, Devereux RB, Fabsitz RR, MacCluer JW. Genetic variation in APOJ, LPL, and TNFRSF10B affects plasma fatty acid distribution in Alaskan Eskimos. The American journal of clinical nutrition. 2010 Jun 1;91(6):1574-83.

[53] Lanktree MB, Anand SS, Yusuf S, Hegele RA. Replication of genetic associations with plasma lipoprotein traits in a multiethnic sample [S]. Journal of lipid research. 2009 Jul 1;50(7):1487-96.

[54] Boes E, Coassin S, Kollerits B, Heid IM, Kronenberg F. Genetic-epidemiological evidence on genes associated with HDL cholesterol levels: a systematic in-depth review. Experimental gerontology. 2009 Mar 1;44(3):136-60.

[55] Arora GP, Åkerlund M, Brøns C, Moen GH, Wasenius NS, Sommer C, Jenum AK, Almgren P, Thaman RG, Orho-Melander M, Eriksson J. Phenotypic and genotypic differences between Indian and Scandinavian women with gestational diabetes mellitus. Journal of internal medicine. 2019 Aug;286(2):192-206.

[56] Arora GP, Almgren P, Brøns C, Thaman RG, Vaag AA, Groop L, Prasad RB. Association between genetic risk variants and glucose intolerance during pregnancy in north Indian women. BMC medical genomics. 2018 Dec;11(1):1-0.

[57] Li-Gao R, Wakil SM, Meyer BF, Dzimiri N, Mook-Kanamori DO. Replication of Type 2 diabetes-associated variants in a Saudi Arabian population. Physiological genomics. 2018 Apr 1;50(4):296-7.

[58] Langlois C, Abadi A, Peralta-Romero J, Alyass A, Suarez F, Gomez-Zamudio J, Burguete-Garcia AI, Yazdi FT, Cruz M, Meyre D. Evaluating the transferability of 15 European-derived fasting plasma glucose SNPs in Mexican children and adolescents. Scientific reports. 2016 Oct 26;6(1):1-8.

[59] O′Beirne SL, Salit J, Rodriguez-Flores JL, Staudt MR, Abi Khalil C, Fakhro KA, Robay A, Ramstetter MD, Al-Azwani IK, Malek JA, Zirie M. Type 2 diabetes risk allele loci in the Qatari population. PloS one. 2016 Jul 6;11(7):e0156834.

[60] Machicao F, Peter A, Machann J, Königsrainer I, Böhm A, Lutz SZ, Heni M, Fritsche A, Schick F, Königsrainer A, Stefan N. Glucose-raising polymorphisms in the human clock gene cryptochrome 2 (CRY2) affect hepatic lipid content. PloS one. 2016 Jan 4;11(1):e0145563.

[61] Dashti HS, Follis JL, Smith CE, Tanaka T, Garaulet M, Gottlieb DJ, Hruby A, Jacques PF, Kiefte-de Jong JC, Lamon-Fava S, Scheer FA. Gene-environment interactions of circadian-related genes for cardiometabolic traits. Diabetes Care. 2015 Aug 1;38(8):1456-66.

[62] Renström F, Koivula RW, Varga TV, Hallmans G, Mulder H, Florez JC, Hu FB, Franks PW. Season-dependent associations of circadian rhythm-regulating loci (CRY1, CRY2 and MTNR1B) and glucose homeostasis: the GLACIER Study. Diabetologia. 2015 May;58(5):997-1005.

[63] Mirzaei K, Xu M, Qi Q, De Jonge L, Bray GA, Sacks F, Qi L. Variants in glucose-and circadian rhythm–related genes affect the response of energy expenditure to weight-loss diets: the POUNDS LOST Trial. The American journal of clinical nutrition. 2014 Feb 1;99(2):392-9.

[64] Borglykke A, Grarup N, Sparsø T, Linneberg A, Fenger M, Jeppesen J, Hansen T, Pedersen O, Jørgensen T. Genetic Variant SCL2A2 Is Associated with Risk of Cardiovascular Disease–Assessing the Individual and Cumulative Effect of 46 Type 2 Diabetes Related Genetic Variants. PLoS One. 2012 Nov 21;7(11):e50418.

[65] Florez JC, Jablonski KA, McAteer JB, Franks PW, Mason CC, Mather K, Horton E, Goldberg R, Dabelea D, Kahn SE, Arakaki RF. Effects of genetic variants previously associated with fasting

glucose and insulin in the Diabetes Prevention Program.

[66] Rees SD, Hydrie MZ, O'Hare JP, Kumar S, Shera AS, Basit A, Barnett AH, Kelly MA. Effects of 16 genetic variants on fasting glucose and type 2 diabetes in South Asians: ADCY5 and GLIS3 variants may predispose to type 2 diabetes. PloS one. 2011 Sep 20;6(9):e24710.

[67] Kanoni S, Nettleton JA, Hivert MF, Ye Z, Van Rooij FJ, Shungin D, Sonestedt E, Ngwa JS, Wojczynski MK, Lemaitre RN, Gustafsson S. Total zinc intake may modify the glucose-raising effect of a zinc transporter (SLC30A8) variant: a 14-cohort meta-analysis. Diabetes. 2011 Sep 1;60(9):2407-16.

[68] Liu C, Li H, Qi L, Loos RJ, Qi Q, Lu L, Gan W, Lin X. Variants in GLIS3 and CRY2 are associated with type 2 diabetes and impaired fasting glucose in Chinese Hans. PLoS One. 2011 Jun 29;6(6):e21464.

[69] Barker A, Sharp SJ, Timpson NJ, Bouatia-Naji N, Warrington NM, Kanoni S, Beilin LJ, Brage S, Deloukas P, Evans DM, Grontved A. Association of genetic Loci with glucose levels in childhood and adolescence: a meta-analysis of over 6,000 children. Diabetes. 2011 Jun 1;60(6):1805-12.

[70] Ramos E, Chen G, Shriner D, Doumatey A, Gerry NP, Herbert A, Huang H, Zhou J, Christman MF, Adeyemo A, Rotimi C. Replication of genome-wide association studies (GWAS) loci for fasting plasma glucose in African-Americans. Diabetologia. 2011 Apr;54(4):783-8.

[71] Hu C, Zhang R, Wang C, Wang J, Ma X, Hou X, Lu J, Yu W, Jiang F, Bao Y, Xiang K. Variants from GIPR, TCF7L2, DGKB, MADD, CRY2, GLIS3, PROX1, SLC30A8 and IGF1 are associated with glucose metabolism in the Chinese. PloS one. 2010 Nov 17;5(11):e15542.

[72] Renström F, Shungin D, Johansson I, MAGIC Investigators, Florez JC, Hallmans G, Hu FB, Franks PW. Genetic predisposition to long-term nondiabetic deteriorations in glucose homeostasis: Ten-year follow-up of the GLACIER study. Diabetes. 2011 Jan 1;60(1):345-54.

[73] Ojeda DA, Perea CS, Niño CL, Gutiérrez RM, López-León S, Arboleda H, Camargo A, Adan A, Forero DA. A novel association of two non-synonymous polymorphisms in PER2 and PER3 genes with specific diurnal preference subscales. Neuroscience letters. 2013 Oct 11;553:52-6.

[74] Lee HJ, Kim L, Kang SG, Yoon HK, Choi JE, Park YM, Kim SJ, Kripke DF. PER2 variation is associated with diurnal preference in a Korean young population. Behavior genetics. 2011 Mar;41(2):273-7.

[75] Lesicka M, Jabłońska E, Wieczorek E, Pepłońska B, Gromadzińska J, Seroczyńska B, Kalinowski L, Skokowski J, Reszka E. Circadian gene polymorphisms associated with breast cancer susceptibility. International journal of molecular sciences. 2019 Jan;20(22):5704.

[76] Jennysdotter Olofsgård F, Ran C, Fourier C, Wirth C, Sjöstrand C, Waldenlind E, Steinberg A, Belin AC. PER Gene Family Polymorphisms in Relation to Cluster Headache and Circadian Rhythm in Sweden. Brain Sciences. 2021 Aug;11(8):1108.

[77] Dan YL, Zhao CN, Mao YM, Wu Q, He YS, Hu YQ, Xiang K, Yang XK, Sam NB, Wu GC, Pan HF. Association of PER2 gene single nucleotide polymorphisms with genetic susceptibility to systemic lupus erythematosus. Lupus. 2021 Apr;30(5):734-40.

[78] Liu JJ, Sudic Hukic D, Forsell Y, Schalling M, Ösby U, Lavebratt C. Depression-associated ARNTL and PER2 genetic variants in psychotic disorders. Chronobiology International. 2015 Apr 21;32(4):579-84.

[79] Lee H, Nah SS, Chang SH, Kim HK, Kwon JT, Lee S, Cho IH, Lee SW, Kim YO, Hong SJ, Kim HJ. PER2 is downregulated by the LPS-induced inflammatory response in synoviocytes in rheumatoid arthritis and is implicated in disease susceptibility. Molecular medicine reports. 2017 Jul 1;16(1):422-8.

[80] Armstrong TS, Vera E, Zhou R, Acquaye AA, Sullaway CM, Berger AM, Breton G, Mahajan A, Wefel JS, Gilbert MR, Bondy M. Association of genetic variants with fatigue in patients with

malignant glioma. Neuro-Oncology Practice. 2018 May 10;5(2):122-8.

[81] Škrlec I, Milić J, Heffer M, Wagner J, Peterlin B. Circadian clock genes and circadian phenotypes in patients with myocardial infarction. Advances in medical sciences. 2019 Sep 1;64(2):224-9.

[82] Benna C, Rajendran S, Spiro G, Tropea S, Del Fiore P, Rossi CR, Mocellin S. Associations of clock genes polymorphisms with soft tissue sarcoma susceptibility and prognosis. Journal of translational medicine. 2018 Dec;16(1):1-1.

[83] Karantanos T, Theodoropoulos G, Gazouli M, Vaiopoulou A, Karantanou C, Stravopodis DJ, Bramis K, Lymperi M, Pektasidis D. Association of the clock genes polymorphisms with colorectal cancer susceptibility. Journal of surgical oncology. 2013 Dec;108(8):563-7.

[84] Garcia-Rios A, Perez-Martinez P, Delgado-Lista J, Phillips CM, Gjelstad IM, Wright JW, Karlström B, Kie´ c-Wilk B, van Hees AM, Helal O, Polus A. A Period 2 genetic variant interacts with plasma SFA to modify plasma lipid concentrations in adults with metabolic syndrome. The Journal of nutrition. 2012 Jul 1;142(7):1213-8.

[85] Song HM, Cho CH, Lee HJ, Moon JH, Kang SG, Yoon HK, Park YM, Kim L. Association of CLOCK, ARNTL, PER2, and GNB3 polymorphisms with diurnal preference in a Korean population. Chronobiology international. 2016 Nov 25;33(10):1455-63.

[86] Chen H, Ding X, Ding E, Chen M, Wang H, Yang G, Zhu B. A missense variant rs2585405 in clock gene PER1 is associated with the increased risk of noise-induced hearing loss in a Chinese occupational population. BMC medical genomics. 2021 Dec;14(1):1-2.

[87] Shirai Y, Fujita Y, Hashimoto R, Ohi K, Yamamori H, Yasuda Y, Ishima T, Suganuma H, Ushida Y, Takeda M, Hashimoto K. Dietary intake of sulforaphane-rich broccoli sprout extracts during juvenile and adolescence can prevent phencyclidine-induced cognitive deficits at adulthood. PLoS One. 2015 Jun 24;10(6):e0127244.

[88] Acosta-Herrera M, Pino-Yanes M, Blanco J, Ballesteros JC, Ambrós A, Corrales A, Gandía F, Subirá C, Domínguez D, Baluja A, Añón JM. Common variants of NFE2L2 gene predisposes to acute respiratory distress syndrome in patients with severe sepsis. Critical care. 2015 Dec;19(1):1-8.

[89] Chen S, Pan H, Chen Y, Lu L, He X, Chen H, Chen R, Zhan S, Tang S. Association between genetic polymorphisms of NRF2, KEAP1, MAFF, MAFK and anti-tuberculosis drug-induced liver injury: a nested case-control study. Scientific reports. 2019 Oct 4;9(1):1-9.

[90] Figarska SM, Vonk JM, Boezen HM. NFE2L2 polymorphisms, mortality, and metabolism in the general population. Physiological genomics. 2014 Jun 15;46(12):411-7.

[91] Siedlinski M, Postma DS, Boer JM, van der Steege G, Schouten JP, Smit HA, Boezen HM. Level and course of FEV 1 in relation to polymorphisms in NFE2L2 and KEAP1 in the general population. Respiratory research. 2009 Dec;10(1):1-2.

[92] Terracciano A, Sanna S, Uda M, Deiana B, Usala G, Busonero F, Maschio A, Scally M, Patriciu N, Chen WM, Distel MA. Genome-wide association scan for five major dimensions of personality. Molecular psychiatry. 2010 Jun;15(6):647-56.

[93] Ci H, Wu N, Su Y. Clock gene modulates roles of OXTR and AVPR1b genes in prosociality. PloS one. 2014 Oct 13;9(10):e109086.

[94] Wang F, Huang G, Shen L, Peng Y, Sha W, Chen ZW, Shen H. Genetics and functional mechanisms of STAT3 polymorphisms in human tuberculosis. Frontiers in Cellular and Infection Microbiology. 2021;11.

[95] Li M, Jiao L, Lyu M, Song J, Bai H, Zhang C, Wu T, Chen X, Ying B. Association of IL27 and STAT3 genetic polymorphism on the susceptibility of tuberculosis in Western Chinese Han population. Infection, Genetics and Evolution. 2020 Sep 1;83:104324.

[96] Li YZ, Zhang L, Liu Q, Bian HT, Cheng WJ. The effect of single nucleotide polymorphisms of STAT3 on epilepsy in children. Eur. Rev. Med. Pharmacol. Sci. 2020 Jan 1;24:837-42.

[97] Stypińska B, Olesińska M, Pawlik A, Paradowska-Gorycka A. Lack of significant association between selected STAT3 polymorphisms and rheumatoid arthritis in the Polish population. Reumatologia. 2018;56(2):73.

[98] Li M, Li F, Li N, Sang J, Fan X, Deng H, Zhang X, Han Q, Lv Y, Liu Z. Association of polymorphism rs1053005 in STAT3 with chronic hepatitis B virus infection in Han Chinese population. BMC medical genetics. 2018 Dec;19(1):1-8.

[99] Zhou F, Cheng L, Qiu LX, Wang MY, Li J, Sun MH, Yang YJ, Wang JC, Jin L, Wang YN, Wei QY. Associations of potentially functional variants in IL-6, JAKs and STAT3 with gastric cancer risk in an eastern Chinese population. Oncotarget. 2016 May 10;7(19):28112.

[100] Xiao L, Muhali FS, Cai TT, Song RH, Hu R, Shi XH, Jiang WJ, Li DF, He ST, Xu J, Zhang JA. Association of single-nucleotide polymorphisms in the STAT3 gene with autoimmune thyroid disease in Chinese individuals. Functional & integrative genomics. 2013 Nov;13(4):455-61.

[101] Slattery ML, Lundgreen A, Kadlubar SA, Bondurant KL, Wolff RK. JAK/STAT/SOCS-signaling pathway and colon and rectal cancer. Molecular carcinogenesis. 2013 Feb;52(2):155-66.

[102] Kwon EM, Salinas CA, Kolb S, Fu R, Feng Z, Stanford JL, Ostrander EA. Genetic polymorphisms in inflammation pathway genes and prostate cancer risk. Cancer Epidemiology and Prevention Biomarkers. 2011 May 1;20(5):923-33.

[103] Davidson SI, Liu Y, Danoy PA, Wu X, Thomas GP, Jiang L, Sun L, Wang N, Han J, Han H, Visscher PM. Association of STAT3 and TNFRSF1A with ankylosing spondylitis in Han Chinese. Annals of the rheumatic diseases. 2011 Feb 1;70(2):289-92.

[104] Phillips CM, Goumidi L, Bertrais S, Field MR, Peloso GM, Shen J, McManus R, Hercberg S, Lairon D, Planells R, Roche HM. Dietary saturated fat modulates the association between STAT3 polymorphisms and abdominal obesity in adults. The Journal of nutrition. 2009 Nov 1;139(11):2011-7.

[105] Yu J, Wang X, Zhu Y, Lu Y, Sun Z. Lack of association between FOXO1 polymorphisms and bacteremia. International journal of clinical and experimental medicine. 2015;8(9):16384.

[106] Park M, Kim SA, Shin J, Joo EJ. Investigation of gene-gene interactions of clock genes for chronotype in a healthy Korean population. Genomics & Informatics. 2020 Dec;18(4).

[107] Kovac U, Jasper EA, Smith CJ, Baer RJ, Bedell B, Donovan BM, Weathers N, Prosenc Zmrzljak U, Jelliffe-Pawlowski LL, Rozman D, Ryckman KK. The association of polymorphisms in circadian clock and lipid metabolism genes with 2nd trimester lipid levels and preterm birth. Frontiers in genetics. 2019 Jun 13;10:540.

[108] Qu F, Qiao Q, Wang N, Ji G, Zhao H, He L, Wang H, Bao G. Genetic polymorphisms in circadian negative feedback regulation genes predict overall survival and response to chemotherapy in gastric cancer patients. Scientific reports. 2016 Mar 1;6(1):1-1.

[109] Zhang Z, Ma F, Zhou F, Chen Y, Wang X, Zhang H, Zhu Y, Bi J, Zhang Y. Functional polymorphisms of circadian negative feedback regulation genes are associated with clinical outcome in hepatocellular carcinoma patients receiving radical resection. Medical Oncology. 2014 Dec 1;31(12):179.

[110] Lesicka M, Jabłońska E, Wieczorek E, Pepłońska B, Gromadzińska J, Seroczyńska B, Kalinowski L, Skokowski J, Reszka E. Circadian gene polymorphisms associated with breast cancer susceptibility. International journal of molecular sciences. 2019 Jan;20(22):5704.

[111] Hida A, Kitamura S, Katayose Y, Kato M, Ono H, Kadotani H, Uchiyama M, Ebisawa T, Inoue Y, Kamei Y, Okawa M. Screening of clock gene polymorphisms demonstrates association of a PER3 polymorphism with morningness–eveningness preference and circadian rhythm sleep disorder. Scientific reports. 2014 Sep 9;4(1):1-6.

[116] Sikora M, Laayouni H, Menendez C, Mayor A, Bardaji A, Sigauque B, Netea MG, Casals F, Bertranpetit J. A targeted association study of immunity genes and networks suggests novel associations with placental malaria infection. PLoS One. 2011 Sep 19;6(9):e24996.

Table S13 Correlations (*r*) between HOMA-IR and clinical parameters in men and women in different age groups

	Women				Men			
	All (n=960)	30s (n=242)	40s (n=447)	50s (n=271)	All (n=444)	30s (n=152)	40s (n=191)	50s (n=101)
BMI	.49***	.54***	.51***	.43***	.54***	.54***	.55***	.53***
ALT	.34***	.34***	.34***	.36***	.36***	.33***	.39***	.33***
Hb	.12***	.08	.12**	.15**	.17***	.19**	.18**	.10
RBC	.24***	.24***	.23***	.26***	.21***	.18**	.17**	.27***
Neutrophil count	.25***	.36***	.26***	.12**	.18***	.32***	.15**	-.02
Lymphocyte count	.16***	.22***	.16***	.09	.09*	.09	.09	.10
NLR	.12***	.18***	.12**	.06	.10**	.21***	.07	-0.05
hsCRP	.33***	.39***	.34***	.24***	.29***	.27***	.33***	.25**
VFA	.47***	.56***	.46***	.44***	.50***	.45***	.55***	.46***

Pearson's correlation coefficient (r) was significant at * p<.05, ** p<.01, *** p<.001. We applied log-normalization before analysis when appropriate.

HOMA-IR, homeostasis model assessment of insulin resistance, BMI, body mass index, ALT, alanine aminotransferase, Hb, hemoglobin, RBC, red blood cells, NLR, neutrophil to lymphocyte ratio, hsCRP, high-sensitivity C-reactive protein, VFA, visceral fat area.

Fig. S1 Correlations (r) between levels of inflammatory markers, trunk fat mass, low-density lipoprotein cholesterol, liver enzymes, hemoglobin, and hematocrit in **a** women (n=960) and **b** men (n=444)

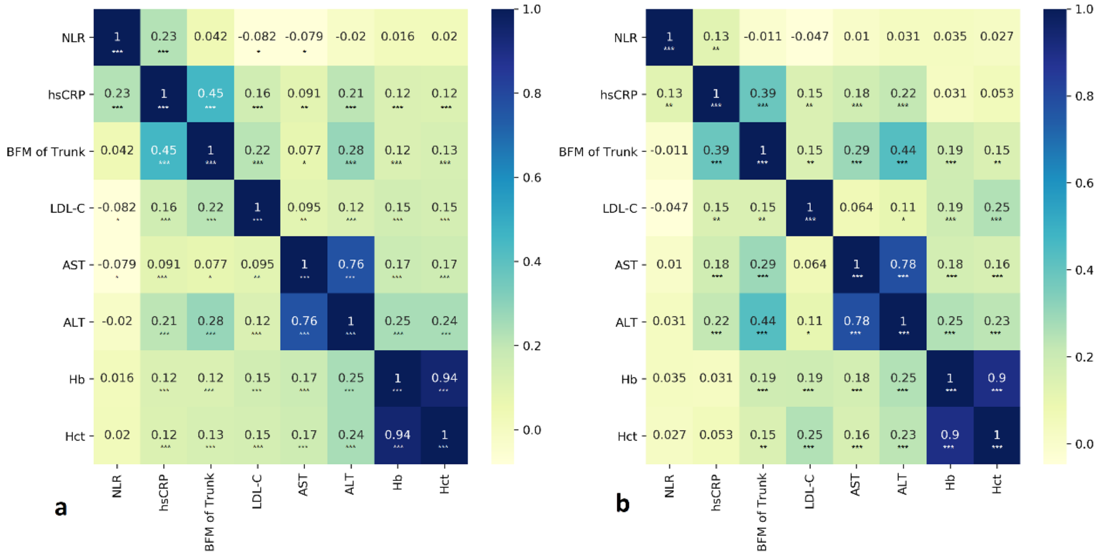

In women, hsCRP was moderately correlated with BFM of the trunk (r=.45, p<.001), and weakly correlated with Hb (r=.12, p<.001) and Hct (r=.12, p<.001). In men, hsCRP was weakly correlated with BFM of the trunk (r=.39, p<.001). There were no correlations between hsCRP and Hb and Hct in men. Meanwhile, in men, BFM of the trunk was moderately correlated with ALT (r=.44, p<.001) and weakly correlated with AST (r=.29, p<.001). In women, BFM of the trunk was weakly correlated with ALT (r=.28, p<.001). There was no correlation between BFM of the trunk and AST in women. NLR, neutrophil to lymphocyte ratio; hsCRP, high sensitivity C-reactive protein; BFM, body fat mass; LDL-C, low-density lipoprotein cholesterol; AST, aspartate aminotransferase; ALT, alanine aminotransferase; Hb, hemoglobin; Hct, hematocrit
